# Risk factors for nephropathy in persons with type 1 diabetes: a population-based study

**DOI:** 10.1007/s00592-022-01863-6

**Published:** 2022-02-24

**Authors:** Shilan Seyed Ahmadi, Aldina Pivodic, Ann-Marie Svensson, Hans Wedel, Björn Rathsman, Thomas Nyström, Johnny Ludvigsson, Marcus Lind

**Affiliations:** 1 Department of Molecular and Clinical Medicine, Institute of Medicine, University of Gothenburg, Gothenburg, Sweden; 2 Department of Internal Medicine, Sahlgrenska University Hospital, Gothenburg, Sweden; 3 Statistiska Konsultgruppen, Gothenburg, Sweden; 4 Department of Clinical Neuroscience, Institute of Neuroscience and Physiology, Sahlgrenska Academy, University of Gothenburg, Gothenburg, Sweden; 5 Centre of Registers in Region Västra Götaland, Gothenburg, Sweden; 6 Department of Health Metrics, Sahlgrenska Academy, University of Gothenburg, Gothenburg, Sweden; 7 Department of Clinical Science and Education, Sachs’ Children and Youth Hospital, Södersjukhuset, Karolinska Institutet, Stockholm, Sweden; 8 Department of Clinical Science and Education, Internal Medicine, Södersjukhuset, Karolinska Institutet, Stockholm, Sweden; 9 Department of Biomedical and Clinical Sciences, Crown Princess Victoria Children’s Hospital, and Division of Paediatrics, Linköping University, Linköping, Sweden; 10 Department of Medicine, NU Hospital Group, Uddevalla, Sweden; 11 Department of Medicine, Uddevalla Hospital, 45180 Uddevalla, Sweden

**Keywords:** Type 1 diabetes, Albuminuria, Lipids, Blood pressure, BMI

## Abstract

**Aims:**

Albuminuria is strongly associated with risk of renal dysfunction, cardiovascular disease and mortality. However, clinical guidelines diverge, and evidence is sparse on what risk factor levels regarding blood pressure, blood lipids and BMI are needed to prevent albuminuria in adolescents and young adults with type 1 diabetes.

**Methods:**

A total of 9347 children and adults with type 1 diabetes [mean age 15.3 years and mean diabetes duration 1.4 years at start of follow-up] from The Swedish National Diabetes Registry were followed from first registration until end of 2017. Levels for risk factors for a risk increase in nephropathy were evaluated, and the gradient of risk per 1 SD (standard deviation) was estimated to compare the impact of each risk factor.

**Results:**

During the follow-up period, 8610 (92.1%) remained normoalbuminuric, 737 (7.9%) individuals developed micro- or macroalbuminuria at any time period of whom 132 (17.9% of 737) individuals developed macroalbuminuria. Blood pressure ≥ 140/80 mmHg was associated with increased risk of albuminuria (*p* ≤ 0.0001), as were triglycerides ≥ 1.0 mmol/L (*p* = 0.039), total cholesterol ≥ 5.0 mmol/L (*p* =  0.0003), HDL < 1.0 mmol/L (*p* = 0.013), LDL 3.5– < 4.0 mmol/L (*p* = 0.020), and BMI ≥ 30 kg/m^2^ (*p* =  0.033). HbA1c was the strongest risk factor for any albuminuria estimated by the measure gradient of risk per 1 SD, followed by diastolic blood pressure, triglycerides, systolic blood pressure, cholesterol and LDL. In patients with HbA1c > 65 mmol/mol (> 8.1%), blood pressure > 140/70 mmHg was associated with increased risk of albuminuria.

**Conclusions:**

Preventing renal complications in adolescents and young adults with type 1 diabetes need avoidance at relatively high levels of blood pressure, blood lipids and BMI, whereas very tight control is not associated with further risk reduction. For patients with long-term poor glycaemic control, stricter blood pressure control is advocated.

**Supplementary Information:**

The online version contains supplementary material available at 10.1007/s00592-022-01863-6.

## Introduction

Diabetic nephropathy (DN), also known as diabetic kidney disease, occurs in 15–40% of all persons with type 1 diabetes. DN is characterised by pathological urinary albumin excretion, glomerular lesions, and loss of glomerular filtration rate [1]. Microalbuminuria has been established as an early marker of progressive kidney disease, and macroalbuminuria an essential risk factor for cardiovascular disease, including stroke, atrial fibrillation, heart failure and mortality [2–4].

The Diabetes Control and Complication Trial (DCCT) and the follow-up Epidemiology of Diabetes Interventions and Complications (EDIC) demonstrated benefits of reducing HbA1c and decreasing the risk of DN with intensive therapy compared with conventional therapy [5, 6]. Blood pressure, dyslipidaemia, diabetes duration and albumin excretion rate have also been shown to be associated with progression of DN [7–16]. Recommendations regarding blood pressure diverge in guidelines from European Society of Cardiology (ESC), American College of Cardiology (ACC)/American Heart Association (AHA) and American Diabetes Association (ADA) [17–19]. Additionally, for blood lipid managements, evidence remains incomplete regarding adults with type 1 diabetes < 40 years old, as earlier studies have mainly been performed in older patients with long diabetes duration [9–14]. To fully understand the impact of a risk factor for development of diabetes complications, it is essential to have information from diagnosis of diabetes and onwards in large, unselected population-based patient cohorts. Correct estimations of risk factors are essential for prognosis, but also for resource allocation and avoiding adverse events from unnecessary medications.

Recently, we found in a large patient cohort, following patients from diagnosis and onwards, that risks of nephropathy gradually increased with HbA1c higher than 52 mmol/mol (7.0%) and no further risk reductions were seen at HbA1c < 48 mmol/mol (6.5%) [20]. In this population-based cohort study using paediatric and adult registries in Sweden, we aimed to evaluate what levels of blood lipids, blood pressure and BMI in persons with type 1 diabetes are related to risk of nephropathy. We also aimed to rank HbA1c, blood lipids, blood pressure, body mass index (BMI), and smoking regarding their risk contribution to development of DN.

## Methods

We conducted a registry-based observational cohort study. Ethical approval was obtained from the Regional Ethical Review Board in Gothenburg, Sweden.

### Data sources

Data were obtained from the paediatric registry, SWEDIABKIDS, and the adult registry, The Swedish National Diabetes Registry (NDR), which started in the years 2000 and 1996, respectively. The registries recently merged and include information on risk factors and complications. For inclusion in the registries, patients and/or guardians provide their informed consent. More than 95% of Swedish children and adolescents with type 1 diabetes are registered in the SWEDIABKIDS, the registries have included data on almost all children and adolescents with diabetes in Sweden [21]. In this study, information from NDR from 1998 onwards and for SWEDIABKIDS from 2000 onwards were included.

### Study population

Current evaluations were performed from a recently used study cohort [20]. A total of 9347 persons with type 1 diabetes were included and followed up during period 1 January 1998 to 31 December 2017. Type 1 diabetes was defined as treatment with insulin and diagnosis < 30 years of age, and this definition has been validated in 97% of cases [22]. To be included, children should have a clinical diagnosis of type 1 diabetes in the register. HLA and diabetes-related autoantibodies are determined in all children with newly diagnosed diabetes. In brief, criteria for inclusion in the study were children and adults who had their type 1 diabetes- diagnosis for < 5 years when first recorded in the registries. In addition, a minimum of 4 visits with non-missing values during a minimum of 8-year follow-up from diagnosis were required.

### Study procedures

Variables assessed were HbA1c (glycated haemoglobin), BMI (body mass index), low-density lipoprotein (LDL), high-density lipoprotein (HDL), cholesterol, triglycerides, systolic blood pressure (SBP), diastolic blood pressure (DBP) and smoking.

As earlier described [20], participants were included in the registers at different time points, whereby subcohorts were categorized by follow-up time from diabetes diagnosis to evaluate risk factors for a certain diabetes duration: 8–9, 10–11, 12–13, 14–15, and 16–20 years. This approach was used to standardize the diabetes duration which is essential for the development of complications. Further, conventional approaches of evaluating time to event by time-updated models could not be used since hazard ratios were not constant over time (assumption of proportional hazards was not fulfilled). We followed participants from the first observation until the end of each subcohort if having a registration regarding albuminuria (normo-, micro- or macroalbuminuria). The primary analysis was a pooled analysis using all the five different subcohorts described above. Two separate analyses were performed for evaluating risk factors for any albuminuria (micro- or macroalbuminuria) and macroalbuminuria, respectively.

Microalbuminuria was defined as two positive tests from three samples taken within 1 year, with an albumin/creatinine ratio of 3–30 mg/mmol (30–300 mg/g) or U-albumin of 20–200 μg/min (20–300 mg/L). Macroalbuminuria was defined as albumin/creatinine ratio > 30 mg/mmol. If the first estimation of elevated albumin/creatinine ratio was registered as macroalbuminuria, this event was included in both the microalbuminuria and macroalbuminuria analyses.

According to instructions to the clinics, the registered blood pressure (SBP and DBP) was the mean value of two supine readings with a cuff of appropriate size and after at least 5 min of rest. HbA1c values were measured in mmol/mol and were also converted to levels in % according to the National Glycohaemoglobin Standardization Program for dual reporting [23]. Laboratory methods at participating care units for analysing HbA1c level and albuminuria are regularly checked with central reference samples of HbA1c and albuminuria to ensure high accuracy [24].

SBP, DBP, LDL, HDL, triglycerides, total cholesterol, BMI and HbA1c were evaluated as mean of longitudinally collected values, as continuous variables in relation to any albuminuria and to macroalbuminuria. Nonlinear effects were studied by analysing categories of the continuous variables; SBP categories (< 110 mmHg, 110– < 120 mmHg, 120– < 130 mmHg, 130– < 140 mmHg, ≥ 140 mmHg), DBP categories (< 60 mmHg, 60– < 70 mmHg, 70– < 80 mmHg, 80– < 85 mmHg, ≥ 85 mmHg), BMI categories (< 18.5 kg/m^2^, 18.5– < 25 kg/m^2^, 25– < 30 kg/m^2^, 30– < 35 kg/m^2^, ≥ 35 kg/m^2^), LDL categories (< 2.0 mmol/l, 2.0– < 2.5 mmol/L, 2.5– < 3.0 mmol/L, 3.0– < 3.5 mmol/L, 3.5– < 4.0 mmol/L, ≥ 4.0 mmol/L), HDL categories (< 1.0 mmol/L, 1.0– < 1.5 mmol/L, 1.5– < 2.0 mmol/L, ≥ 2.0 mmol/L), triglycerides categories (< 0.5 mmol/L, 0.5– < 1.0 mmol/L, 1.0– < 1.5 mmol/L, 1.5–2.0 mmol/L, ≥ 2.0 mmol/L), cholesterol categories (< 4.0 mmol/L, 4.0– < 5.0 mmol/L, 5.0– < 6.0 mmol/L, ≥ 6.0 mmol/L) and smoking (no versus yes). Odds ratios were described for the following changes in the risk factors: SBP (10 mmHg), DBP (5 mmHg), blood lipid levels (1 mmol/L), BMI (5 kg/m^2^) and HbA1c (10 mmol/mol, 1%). To rank the relative contribution of the risk factors, the metric gradient of risk per one SD was used [25] determining how much the risk of any and macroalbuminuria changes per one SD change in the risk factor. Additionally, sensitivity analyses on all persons > 13 years of age at diabetes diagnosis were performed for the above-mentioned analyses. The impact of risk factor categories on any albuminuria and macroalbuminuria were further analysed after subgrouping for a mean level of HbA1c above or below 65 mmol/mol (8.1%) during follow-up.

### Statistical analysis

Numbers and percentages (with 95% confidence intervals) of complications were expressed for each category of SBP, DBP, BMI, LDL, HDL, triglycerides and cholesterol. Generalised estimating equations modelling was used to estimate the relation between the risk factors and endpoints for diabetic nephropathy, adjusting for within patient correlation for repeated data over the 5 follow-up cohorts. This method allows more than one observation per patient. Unstructured covariance matrix was used. The use of binomial distribution with logit link function resulted in odds ratios (95% confidence intervals) as risk estimates. Analyses were performed for continuous variables and by categories of HbA1c, SBP, DBP, BMI, LDL, HDL, triglycerides and cholesterol, and for smoking. Risk factors were analysed adjusted for age and sex and additionally for mean HbA1c. Mean HbA1c was analysed adjusted for age and sex and additionally for SBP, BMI, triglycerides, cholesterol and smoking.

Pearson correlation was calculated when analysing association between two continuous variables.

No data were imputed. Tests were two-tailed and conducted at 0.05 significance level. All statistical programming was performed using SAS Software version 9.4 (SAS Institute, Cary, NC).

## Results

### Patient characteristics

A total of 9347 persons with type 1 diabetes were included. Patient characteristics are shown in Table 1 for the total cohort and for patients without albuminuria during follow-up and with micro- and/or macroalbuminuria at any time period during follow-up, respectively.

**Table 1:** Patient characteristics

Variable	All patients (*n*=9347)	All patients with no albuminuria (*n*=8610)	All patients with micro- or macroalbuminuria in any time period (*n*=737)	All patients with no macroalbuminuria (*n*=9215)	All patients with macroalbuminuria in any time period (*n*=132)
*Sex*					
Male	5244 (56.1%)	4883 (56.7%)	361 (49.0%)	5184 (56.3%)	60 (45.5%)
Female	4103 (43.9%)	3727 (43.3%)	376 (51.0%)	4031 (43.7%)	72 (54.5%)
Age at first visit	15.3 (7.9) *n*=9347	15.2 (7.9) *n*=8610	16.0 (7.9) *n*=737	15.2 (7.9) *n*=9215	17.2 (8.4) *n*=132
Diabetes onset year	2003 (4) *n*=9347	2003 (4) *n*=8610	2002 (4) *n*=737	2003 (4) *n*=9215	2002 (4) *n*=132
HbA1c mean (%) for longest follow-up	7.94 (1.04) *n*=9347	7.89 (1.01) *n*=8610	8.49 (1.27) *n*=737	7.93 (1.03) *n*=9215	8.57 (1.40) *n*=132
HbA1c mean (mmol/mol) for longest follow-up	63.2 (11.4) *n*=9347	62.7 (11.0) *n*=8610	69.3 (13.9) *n*=737	63.1 (11.3) *n*=9215	70.1 (15.3) *n*=132
*HbA1c mean category for longest follow-up*					
<48 mmol/mol	667 (7.1%)	642 (7.5%)	25 (3.4%)	662 (7.2%)	5 (3.8%)
48-52 mmol/mol	932 (10.0%)	889 (10.3%)	43 (5.8%)	924 (10.0%)	8 (6.1%)
53-57 mmol/mol	1511 (16.2%)	1419 (16.5%)	92 (12.5%)	1498 (16.3%)	13 (9.8%)
58-70 mmol/mol	3963 (42.4%)	3701 (43.0%)	262 (35.5%)	3914 (42.5%)	49 (37.1%)
>70 mmol/mol	2274 (24.3%)	1959 (22.8%)	315 (42.7%)	2217 (24.1%)	57 (43.2%)
SBP mean (mmHg) for longest follow-up	116.7 (8.8) *n*=9330	116.5 (8.6) *n*=8594	118.2 (10.0) *n*=736	116.6 (8.7) *n*=9198	119.1 (12.0) *n*=132
*SBP mean category for longest follow-up*					
<110 mmHg	2048 (22.0%)	1911 (22.2%)	137 (18.6%)	2021 (22.0%)	27 (20.5%)
110-<120 mmHg	4129 (44.3%)	3812 (44.4%)	317 (43.1%)	4074 (44.3%)	55 (41.7%)
120-<130 mmHg	2490 (26.7%)	2286 (26.6%)	204 (27.7%)	2461 (26.8%)	29 (22.0%)
130-<140 mmHg	567 (6.1%)	512 (6.0%)	55 (7.5%)	556 (6.0%)	11 (8.3%)
>=140 mmHg	96 (1.0%)	73 (0.8%)	23 (3.1%)	86 (0.9%)	10 (7.6%)
DBP mean (mmHg) for longest follow-up	70.1 (5.9) *n*=9330	69.9 (5.8) *n*=8594	71.7 (6.8) *n*=736	70.0 (5.8) *n*=9198	72.4 (8.2) *n*=132
*DBP mean category for longest follow-up*					
<60 mmHg	317 (3.4%)	298 (3.5%)	19 (2.6%)	314 (3.4%)	3 (2.3%)
60-<70 mmHg	4425 (47.4%)	4133 (48.1%)	292 (39.7%)	4368 (47.5%)	57 (43.2%)
70-<80 mmHg	4086 (43.8%)	3741 (43.5%)	345 (46.9%)	4036 (43.9%)	50 (37.9%)
80-<85 mmHg	397 (4.3%)	347 (4.0%)	50 (6.8%)	388 (4.2%)	9 (6.8%)
>=85 mmHg	105 (1.1%)	75 (0.9%)	30 (4.1%)	92 (1.0%)	13 (9.8%)
BMI mean (kg/m2) for longest follow-up	22.9 (4.0) *n*=9316	22.9 (4.0) *n*=8583	23.6 (4.9) *n*=733	22.9 (4.0) *n*=9185	23.9 (5.0) *n*=131
*BMI mean category for longest follow-up*					
<18.5 kg/m^2	1038 (11.1%)	961 (11.2%)	77 (10.5%)	1025 (11.2%)	13 (9.9%)
18.5-<25 kg/m^2	5880 (63.1%)	5450 (63.5%)	430 (58.7%)	5804 (63.2%)	76 (58.0%)
25-<30 kg/m^2	1917 (20.6%)	1762 (20.5%)	155 (21.1%)	1889 (20.6%)	28 (21.4%)
30-<35 kg/m^2	383 (4.1%)	336 (3.9%)	47 (6.4%)	373 (4.1%)	10 (7.6%)
>=35 kg/m^2	98 (1.1%)	74 (0.9%)	24 (3.3%)	94 (1.0%)	4 (3.1%)
HDL mean (mmol/L) for longest follow-up	1.52 (0.38) *n*=8534	1.52 (0.38) *n*=7844	1.47 (0.38) *n*=690	1.52 (0.38) *n*=8413	1.48 (0.42) *n*=121
*HDL mean category for longest follow-up*					
<1.0 mmol/L	383 (4.5%)	332 (4.2%)	51 (7.4%)	372 (4.4%)	11 (9.1%)
1.0-<1.5 mmol/L	4026 (47.2%)	3681 (46.9%)	345 (50.0%)	3971 (47.2%)	55 (45.5%)
1.5-<2.0 mmol/L	3233 (37.9%)	2998 (38.2%)	235 (34.1%)	3191 (37.9%)	42 (34.7%)
>=2.0 mmol/L	892 (10.5%)	833 (10.6%)	59 (8.6%)	879 (10.4%)	13 (10.7%)
LDL mean (mmol/L) for longest follow-up	2.53 (0.69) *n*=8539	2.51 (0.68) *n*=7846	2.65 (0.73) *n*=693	2.52 (0.68) *n*=8422	2.72 (0.79) *n*=117
*LDL mean category for longest follow-up*					
<2.0 mmol/L	1832 (21.5%)	1709 (21.8%)	123 (17.7%)	1812 (21.5%)	20 (17.1%)
2.0-<2.5 mmol/L	2617 (30.6%)	2427 (30.9%)	190 (27.4%)	2589 (30.7%)	28 (23.9%)
2.5-<3.0 mmol/L	2191 (25.7%)	2013 (25.7%)	178 (25.7%)	2159 (25.6%)	32 (27.4%)
3.0-<3.5 mmol/L	1175 (13.8%)	1061 (13.5%)	114 (16.5%)	1155 (13.7%)	20 (17.1%)
3.5-<4.0 mmol/L	501 (5.9%)	440 (5.6%)	61 (8.8%)	490 (5.8%)	11 (9.4%)
>=4.0 mmol/L	223 (2.6%)	196 (2.5%)	27 (3.9%)	217 (2.6%)	6 (5.1%)
Cholesterol mean (mmol/L) for longest follow-up	4.49 (0.78) *n*=8707	4.48 (0.77) *n*=8010	4.68 (0.85) *n*=697	4.49 (0.78) *n*=8587	4.89 (0.99) *n*=120
*Cholesterol mean category for longest follow-up*					
<4.0 mmol/L	2250 (25.8%)	2107 (26.3%)	143 (20.5%)	2231 (26.0%)	19 (15.8%)
4.0-<5.0 mmol/L	4394 (50.5%)	4074 (50.9%)	320 (45.9%)	4344 (50.6%)	50 (41.7%)
5.0-<6.0 mmol/L	1721 (19.8%)	1533 (19.1%)	188 (27.0%)	1684 (19.6%)	37 (30.8%)
>=6.0 mmol/L	342 (3.9%)	296 (3.7%)	46 (6.6%)	328 (3.8%)	14 (11.7%)
Triglycerides mean (mmol/L) for longest follow-up	1.07 (0.68) *n*=8300	1.04 (0.59) *n*=7630	1.38 (1.24) *n*=670	1.06 (0.64) *n*=8189	1.75 (1.94) *n*=111
*Triglycerides mean category for longest follow-up*					
<0.5 mmol/L	335 (4.0%)	317 (4.2%)	18 (2.7%)	333 (4.1%)	2 (1.8%)
0.5-<1.0 mmol/L	4550 (54.8%)	4267 (55.9%)	283 (42.2%)	4513 (55.1%)	37 (33.3%)
1.0-<1.5 mmol/L	2175 (26.2%)	1980 (26.0%)	195 (29.1%)	2148 (26.2%)	27 (24.3%)
1.5-2.0 mmol/L	687 (8.3%)	613 (8.0%)	74 (11.0%)	670 (8.2%)	17 (15.3%)
>=2.0 mmol/L	553 (6.7%)	453 (5.9%)	100 (14.9%)	525 (6.4%)	28 (25.2%)
*Smoking at any time before for longest follow-up*					
No	6518 (77.8%)	6021 (78.4%)	497 (71.3%)	6436 (78.0%)	82 (65.1%)
Yes	1862 (22.2%)	1662 (21.6%)	200 (28.7%)	1818 (22.0%)	44 (34.9%)

For categorical variables *n* (%) is presented.

For continuous variables Mean (SD)/*n* = is presented.

The proportion of women in the cohort was 43.9%, the mean HbA1c for the longest follow-up was 63.2 ± 11.4 mmol/mol (7.9 ± 1.0%), mean blood pressure 116 ± 9/70 ± 6 mmHg, mean BMI 22.9 ± 4.0 kg/m^2^, mean LDL 2.5 ± 0.7 mmol/L, mean HDL 1.5 ± 0.4 mmol/L, mean cholesterol 4.5 ± 0.8 mmol/L, and mean triglycerides 1.1 ± 0.7 mmol/L. The mean age at first registered visit was 15.3 ± 7.9 years, and the mean duration of diabetes at first registration was 1.4 ± 1.7 years. Median follow-up was 12.0 years (range 8.0–20.0). In Supplemental Table 1, patient characteristics for the different subcohorts are shown.

### Relative contribution of risk factors for DN

Among 9347 children and adults with type 1 diabetes from the Swedish National Diabetes Registry and observed over a median of 12.0 years, 737 (7.9%) developed any albuminuria; 132 out of these 737 (17.9%) developed macroalbuminuria. Levels of risk factors for a significant risk increase in albuminuria were evaluated and ranking of risk factors was estimated (Table 2). For both any albuminuria and macroalbuminuria, HbA1c was the strongest risk factor. For any albuminuria, the remaining risk factors were ranked as follows: DBP, triglycerides, SBP, total cholesterol and LDL. No impact for: HDL, BMI and smoking. For macroalbuminuria, the remaining risk factors were ranked as follows: total cholesterol, SBP, DBP, and triglycerides. No impact existed for: BMI, LDL, HDL and smoking.

**Table 2:** Generalized estimating equation (GEE) models for the impact of risk factors on nephropathy endpoints

			Adjusted for age and sex			Multivariable adjusted*		
Variable	*n* (%) (95% CI) events**	*n* (%) (95% CI) persons with events***	OR (95% CI) per specified unit increase	OR (95% CI) per 1 SD increase	*p*-value	OR (95% CI) per specified unit increase	OR (95% CI) per 1 SD increase	*p*-value
*Microalbuminuria/Macroalbuminuria vs None*								
Mean HbA1c (mmol/mol) (by 10 unit increase)	1027 (6.1%) (5.7%–6.5%)	737 (7.9%) (7.3%–8.4%)	1.55 (1.46–1.66)	1.65 (1.53–1.78)	<.0001	1.49 (1.39–1.61)	1.58 (1.45–1.72)	<.0001
Mean HbA1c (%) (by 1 unit increase)	1027 (6.1%) (5.7%–6.5%)	737 (7.9%) (7.3%–8.4%)	1.62 (1.51–1.73)	1.65 (1.53–1.77)	<.0001	1.55 (1.43–1.68)	1.58 (1.45–1.71)	<.0001
Mean SBP (mmHg) (by 10 unit increase)	1026 (6.1%) (5.7%–6.5%)	736 (7.9%) (7.3%–8.5%)	1.28 (1.16–1.42)	1.24 (1.13–1.35)	<.0001	1.25 (1.12–1.38)	1.21 (1.10–1.32)	<.0001
Mean DBP (mmHg) (by 5 unit increase)	1026 (6.1%) (5.7%–6.5%)	736 (7.9%) (7.3%–8.5%)	1.30 (1.21–1.41)	1.35 (1.24–1.48)	<.0001	1.23 (1.14–1.34)	1.27 (1.16–1.39)	<.0001
Mean BMI (kg/m2) (by 5 unit increase)	1022 (6.1%) (5.7%–6.5%)	733 (7.9%) (7.3%–8.4%)	1.19 (1.06–1.34)	1.15 (1.05–1.25)	0.0031	1.10 (0.98–1.23)	1.07 (0.98–1.17)	0.12
Mean LDL (mmol/L) (by 1 unit increase)	957 (6.2%) (5.8%–6.5%)	689 (8.1%) (7.5%–8.7%)	1.32 (1.18–1.47)	1.21 (1.12–1.31)	<.0001	1.17 (1.05–1.30)	1.11 (1.03–1.20)	0.0056
Mean HDL (mmol/L) (by 1 unit increase)	959 (6.2%) (5.8%–6.6%)	688 (8.1%) (7.5%–8.7%)	0.68 (0.51–0.89)	0.86 (0.77–0.95)	0.0046	0.82 (0.63–1.05)	0.92 (0.84–1.02)	0.12
Mean triglycerides (mmol/L) (by 1 unit increase)	930 (6.2%) (5.8%–6.6%)	668 (8.0%) (7.5%–8.7%)	1.58 (1.44–1.73)	1.35 (1.27–1.44)	<.0001	1.37 (1.25–1.50)	1.23 (1.16–1.31)	<.0001
Mean cholesterol (mmol/L) (by 1 unit increase)	968 (6.1%) (5.8%–6.5%)	695 (8.0%) (7.4%–8.6%)	1.36 (1.24–1.51)	1.28 (1.19–1.39)	<.0001	1.20 (1.09–1.32)	1.16 (1.07–1.25)	0.0002
Smoking at any time before (yes vs no)	975 (6.2%) (5.9%–6.6%)	695 (8.3%) (7.7%–8.9%)	1.42 (1.19–1.69)	1.15 (1.07–1.24)	<.0001	1.05 (0.88–1.26)	1.02 (0.95–1.10)	0.58
*Macroalbuminuria vs None/Microalbuminuria*								
Mean HbA1c (mmol/mol) (by 10 unit increase)	163 (1.0%) (0.8%–1.1%)	132 (1.4%) (1.2%–1.7%)	1.59 (1.35–1.86)	1.69 (1.41–2.03)	<.0001	1.46 (1.22–1.74)	1.54 (1.26–1.88)	<.0001
Mean HbA1c (%) (by 1 unit increase)	163 (1.0%) (0.8%–1.1%)	132 (1.4%) (1.2%–1.7%)	1.66 (1.39–1.98)	1.69 (1.41–2.03)	<.0001	1.51 (1.25–1.83)	1.54 (1.26–1.88)	<.0001
Mean SBP (mmHg) (by 10 unit increase)	163 (1.0%) (0.8%–1.1%)	132 (1.4%) (1.2%–1.7%)	1.47 (1.13–1.90)	1.39 (1.11–1.73)	0.0037	1.40 (1.09–1.80)	1.33 (1.08–1.65)	0.0083
Mean DBP (mmHg) (by 5 unit increase)	163 (1.0%) (0.8%–1.1%)	132 (1.4%) (1.2%–1.7%)	1.36 (1.12–1.65)	1.42 (1.14–1.76)	0.0017	1.28 (1.05–1.56)	1.32 (1.06–1.66)	0.014
Mean BMI (kg/m2) (by 5 unit increase)	162 (1.0%) (0.8%–1.1%)	131 (1.4%) (1.2%–1.7%)	1.15 (0.90–1.47)	1.11 (0.92–1.35)	0.27	1.04 (0.80–1.35)	1.03 (0.84–1.26)	0.78
Mean LDL (mmol/L) (by 1 unit increase)	144 (0.9%) (0.8%–1.1%)	116 (1.4%) (1.1%–1.6%)	1.38 (1.03–1.84)	1.25 (1.02–1.53)	0.031	1.27 (0.98–1.66)	1.18 (0.98–1.42)	0.076
Mean HDL (mmol/L) (by 1 unit increase)	148 (1.0%) (0.8%–1.1%)	120 (1.4%) (1.2%–1.7%)	0.65 (0.32–1.31)	0.84 (0.64–1.11)	0.23	0.89 (0.45–1.74)	0.96 (0.73–1.24)	0.73
Mean triglycerides (mmol/L) (by 1 unit increase)	137 (0.9%) (0.8%–1.1%)	110 (1.3%) (1.1%–1.6%)	1.66 (1.44–1.91)	1.40 (1.27–1.53)	<.0001	1.50 (1.31–1.72)	1.31 (1.20–1.43)	<.0001
Mean cholesterol (mmol/L) (by 1 unit increase)	147 (0.9%) (0.8%–1.1%)	119 (1.4%) (1.1%–1.6%)	1.64 (1.28–2.09)	1.48 (1.22–1.81)	<.0001	1.49 (1.21–1.84)	1.38 (1.16–1.63)	0.0002
Smoking at any time before (yes vs no)	156 (1.0%) (0.8%–1.2%)	125 (1.5%) (1.2%–1.8%)	1.81 (1.23–2.67)	1.28 (1.09–1.50)	0.0027	1.30 (0.87–1.95)	1.11 (0.94–1.31)	0.21

*HbA1c mean adjusted for age, sex, SBP, BMI, triglycerides, cholesterol and smoking. All other variables adjusted for age, sex and HbA1c mean.

**The number of events pooled over all periods.

***The number of individuals with at least one event pooled over all periods.

### Thresholds for risk of DN

The variables were analysed as categorical variables for the impact on any albuminuria endpoints evaluated for different thresholds adjusted for age, sex and mean HbA1c, as a study of potential nonlinear effects.

**Fig. 1:**
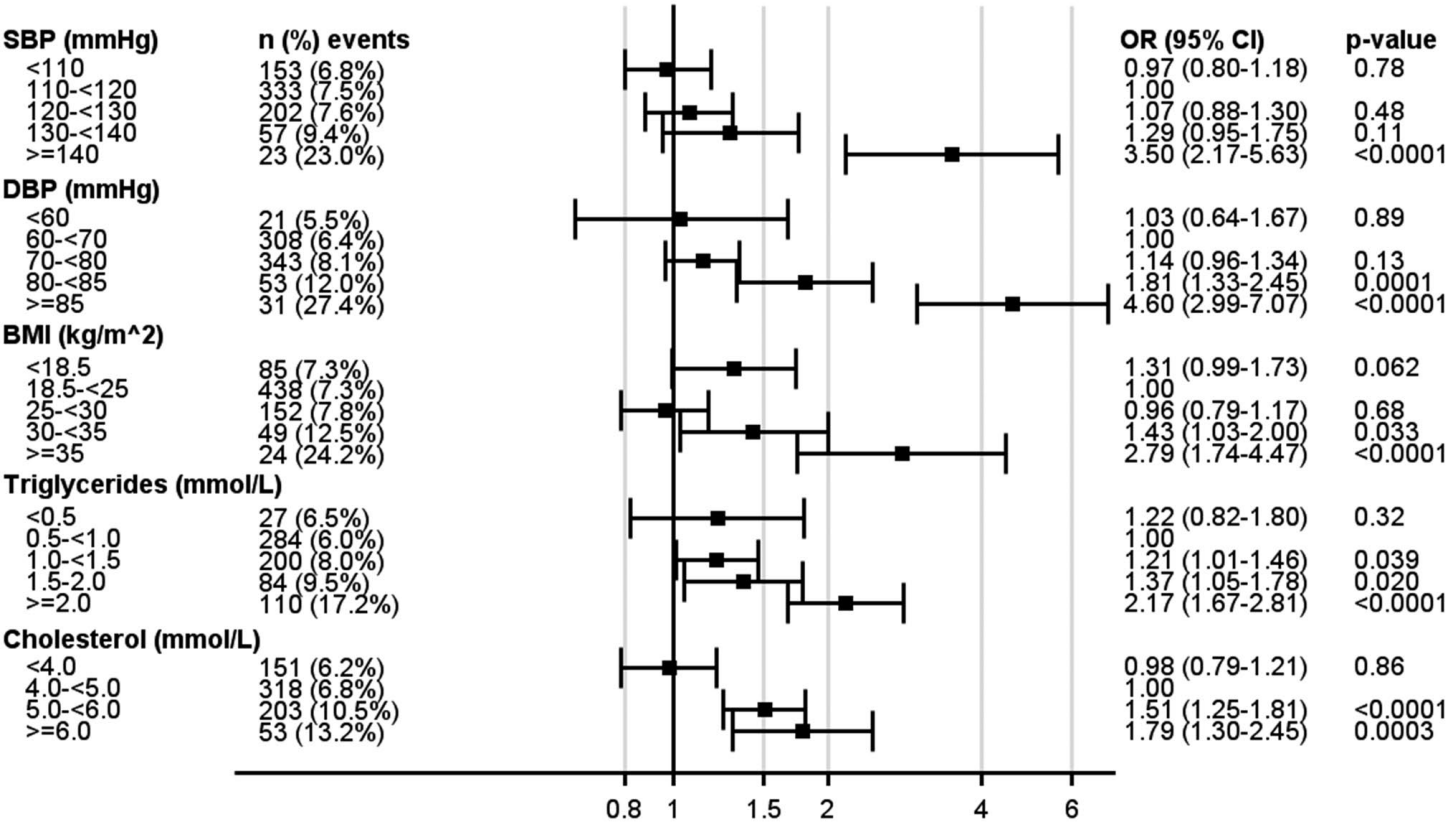
Generalized Estimating Equation (GEE) models for the impact of various variable categories on any albuminuria

**Fig. 2:**
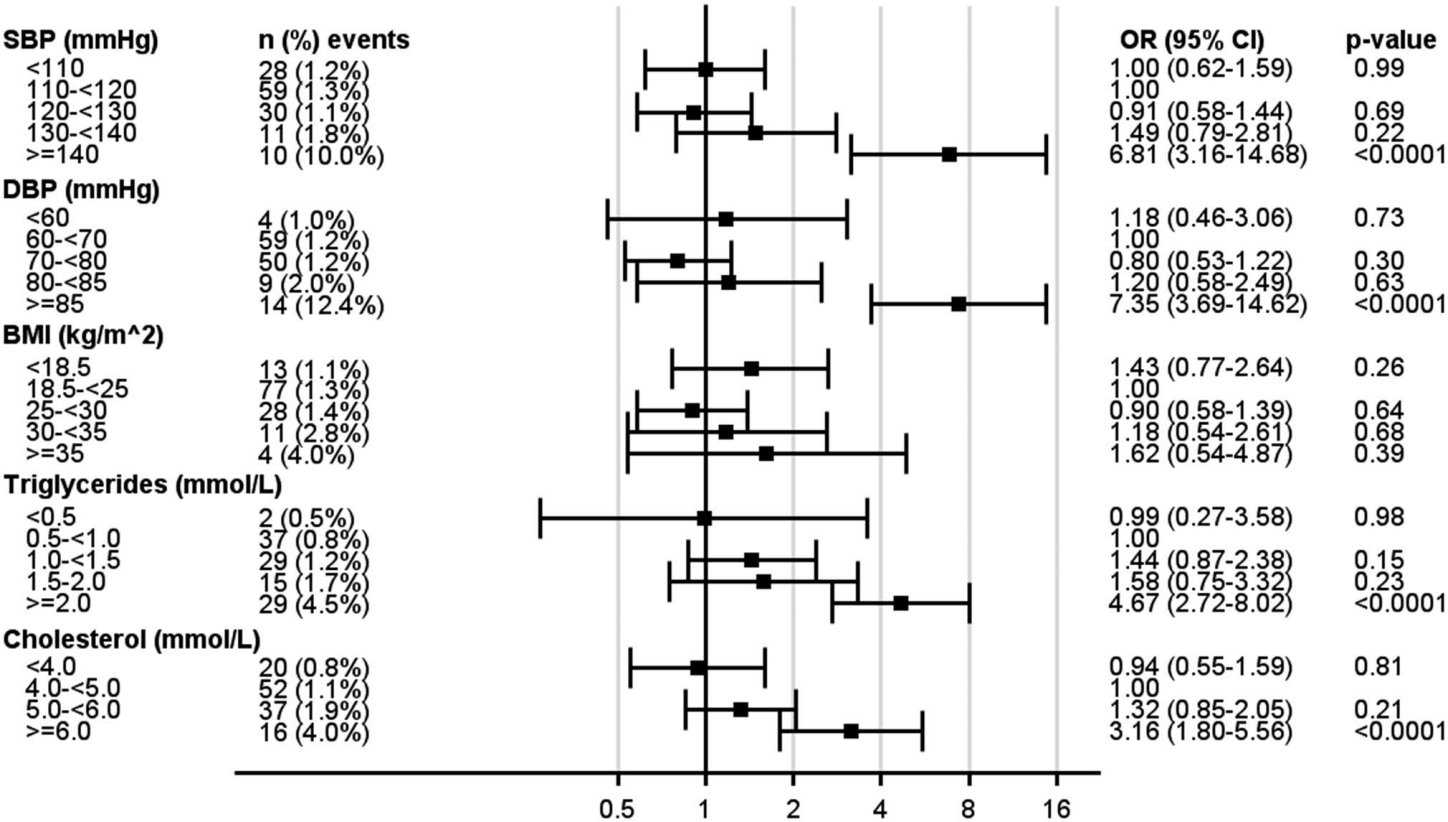
Generalized Estimating Equation (GEE) models for the impact of various variable categories on macroalbuminuria

As seen in Fig. 1, the risk of any albuminuria increased at SBP ≥ 140 mmHg compared with the reference category (110–120 mmHg) and at DBP ≥ 80 mmHg compared with the reference category (60– < 70 mmHg). For macroalbuminuria (Fig. 2), blood pressure ≥ 140/85 mmHg increased the risk*.* For blood lipids, the risk of any albuminuria increased for LDL 3.5– < 4.0 mmol/L, HDL < 1.0 mmol/L, triglycerides 1.0– < 1.5 mmol/L, and cholesterol 5.0– < 6.0 mmol/L compared to each reference category. For BMI, the risk of any albuminuria increased at ≥ 30 kg/m^2^. For macroalbuminuria, mean triglycerides and cholesterol remained significant risk factors for the highest category compared to the respective reference category. Associations between BMI categories and macroalbuminuria, showed numerically higher OR, although not being significant.

In the supplementary appendix, the categorized levels of SBP, DBP, LDL, HDL, triglycerides, cholesterol and BMI analysed for the impact on albuminuria endpoints are shown and, additionally, adjusted for age and sex (Supplemental Table 2).

### Impact of risk factors on subgrouping of HbA1c

The impact of various risk factors on albuminuria was also evaluated based on mean HbA1c levels during follow-up (≤ 65 mmol/mol and > 65 mmol/mol [8.1%]) adjusted for age, sex and mean HbA1c, as shown in Supplemental Table 3. For persons with mean HbA1c > 65 mmol/mol during follow-up, a blood pressure > 140/70 mmHg was associated with increased risk of albuminuria, whereas for persons with HbA1c ≤ 65 mmol/mol, blood pressure > 140/80 mmHg was associated with increased risk.

### Sensitivity analysis

Similar patterns were observed in a sensitivity analysis when patients > 13 years of age at diabetes diagnosis were included regarding their impact on development of any albuminuria. The patient characteristics are shown in the Supplemental Table 4. In brief, 4606 patients were included in the analyses. Numerically, they had a lower mean HbA1c, higher mean blood pressure, comparable mean blood lipids and higher mean BMI at baseline compared to the full cohort population. The same target levels for blood pressure (≥ 140/80 mmHg) and BMI (≥ 30 kg/m^2^) were associated with increased risk of any albuminuria (Supplement Table 5).

As observed in Supplemental Table 6, the impact of continuous risk factors expressed by per specified unit and 1 SD increase on nephropathy endpoints are shown. For any albuminuria, HbA1c followed by DBP, SBP, triglycerides, BMI and HDL remained the significant risk factors. For macroalbuminuria, blood pressure and triglycerides had a significant impact, but not other studied variables.

## Conclusions

In this population-based cohort study, we used both pediatric and adult registries to evaluate the impact of various risk factors on the development of albuminuria in persons with type 1 diabetes. HbA1c was the strongest risk factor for any albuminuria, followed by blood pressure, blood lipids and BMI. No association was seen for smoking. For macroalbuminuria, systolic and diastolic blood pressure, triglycerides and cholesterol remained significant non-glycaemic risk factors for the development of albuminuria. In persons with poor glycaemic control (mean HbA1c > 65 mmol/mol [8.1%]), blood pressure > 140/70 mmHg was associated with increased risk of albuminuria.

In 1993, DCCT showed beneficial effects of reducing HbA1c and decreasing the risk of nephropathy with intensive therapy compared with conventional therapy [5]. In the follow-up EDIC-study, the beneficial effects on albumin excretion and the reduced incidence of hypertension after the DCCT suggested that previous intensive treatment had extended benefit in delaying progression of diabetic nephropathy [6]. The importance of glycaemic control on nephropathy has been shown in several other studies [7–12]. Since then, multiple studies have evaluated other risk factors for progression of albuminuria. Hypertension, dyslipidaemia, and diabetes duration are well-known risk factors, whereas the impact of gender, smoking and BMI have shown diverging results [7–12]. Recently, a study, using the DCCT/EDIC cohort, showed that the risk of macroalbuminuria was associated with sex, blood pressure and lipids (LDL, HDL and triglycerides) [16]. Our results expand upon the results from prior studies by providing data from persons with type 1 diabetes in a population-based contemporary cohort with short diabetes duration at baseline (mean 1.4 years) followed over 8–20 years, as well as what levels of these risk factors increases the risk of albuminuria.

In our study, SBP and DBP were evaluated both as continuous and categorical variables. Both analyses showed that blood pressure has an important impact on the risk of albuminuria. As a continuous variable, an increase in 10 mmHg for mean SBP increased the risk of any albuminuria by 25% and for mean DBP by 35% with an increase in each 5 mmHg. When evaluating SBP and DBP as a categorical variable, levels above 140/80 increased risk of albuminuria. For DBP ≥ 85 mmHg, the OR was increased to 4.6. According to guidelines from ADA and ESC, hypertension is defined as ≥ 140/90 mmHg, whereas ACC/AHA define hypertension as > 130/90 mmHg. Additionally, the guidelines differ in recommendations regarding risk assessment and target goals in adults. Further, the recommendations are mainly based on clinical trials on an older population with or without type 2 diabetes [17–19]. Hence, few high-quality data exist for blood pressure targets in type 1 diabetes from diagnosis and onwards.

Another important risk factor for albuminuria was blood lipids. Our results show that the risk of albuminuria increased at following levels: triglycerides ≥ 1.0 mmol/L, total cholesterol ≥ 5.0 mmol/L, LDL  3.5– < 4.0 mmol/L and HDL < 1.0 mmol/L compared to each group selected reference. Of the blood lipids, triglycerides had the strongest impact on risk of albuminuria after HbA1c and diastolic blood pressure. In a cross-sectional analysis, it was shown that glycaemic control was an important mediator of lipid abnormalities in youth with type 1 diabetes [26]. Additionally, in the FinnDiane study, it was shown that triglyceride and cholesterol levels were associated with incident albuminuria [27]. The mechanisms responsible for the dyslipidaemia in type 1 diabetes remain unclear, but the subcutaneous route of insulin administration, that is responsible for peripheral hyperinsulinemia, may play a role [28]. Another possible explanation is weight gain, and central obesity from a sedentary lifestyle. The individuals with type 1 diabetes and an associated metabolic syndrome have an increased cardiovascular risk compared to other type 1 diabetes patients related to the development of atherogenic lipid profile, in which hypertriglyceridemia is an essential component [29].

In clinical practice, caregivers are more hesitant to treat younger individuals with medications for increased blood pressure and blood lipids. There is a risk of side effects with medications, evidence is less robust due to lack of randomized trials in this patient group and younger individuals have lower adherence to treatment [30]. It is, therefore, important to know at what levels risk factors besides HbA1c really increases the risk of albuminuria. Systolic blood pressure of 130 compared to 140 mmHg has been debated for long periods [31]. This study supports the higher blood pressure level, but at repeated determinations above these values, younger individuals should also get antihypertensive drugs. Additionally, since risk factors can be more difficult to judge among young children, we analysed in a sensitivity analysis patient with diabetes diagnosis after 13 years of age, the same age span as in the DCCT study, with confirming results. In several diseases, strict risk factor targets have been found to be beneficial in prevention to more severe disease, e.g. lower blood lipid targets after myocardial infarction. In the current study, patients with high mean HbA1c (> 8.1%) during follow-up seem to benefit of an even lower diastolic blood pressure of < 70 mmHg. Hence, stricter blood pressure control is indicated in persons with type 1 diabetes with poor glycaemic control.

In the case of statins, these are more commonly used at slightly older ages when the risk of cardiovascular disease increases. With clearly increased LDL and cholesterol levels in younger individuals, one should first exclude familial hypercholesterolemia and then consider medication if dietary advice is not enough [32]. Regarding BMI, previous studies have not found that obesity is a strong risk factor for myocardial infarction and mortality [33]. However, the risk of heart failure, which is significantly more common in type 1 diabetes than in the general population, clearly increases above BMI 30 [34, 35]. In addition, in a study from National Diabetes Audit data, which included individuals with type 1 and type 2 diabetes in UK and Wales, association between obesity and kidney disease was shown [36]. The fact that we see that the risk of albuminuria increases above BMI 30 indicates that an important focus in diabetes care among adolescents and young adults should be to avoid obesity. Furthermore, a weight reduction can contribute to reduced blood pressure in obese patients [37].

A strength of the current study is that patients were followed from close after type 1 diabetes diagnosis (mean diabetes duration 1.4 years at inclusion) over 8–20 years. It is essential to have the complete historical risk factor profile from patients before development of diabetes complications. However, this study was limited by the absence of other factors contributing to albuminuria which were not documented in the registry (e.g., other renal diseases). Since the current study was not randomized, residual confounding cannot be excluded. However, since randomized trials are lacking in the current age group, this first large population-based study following non-glycaemic risk factors from diagnosis of type 1 diabetes is an important contribution to the evidence for risk factor targets in adolescents and young adults with type 1 diabetes.

In this analysis of nationwide registers, it was shown that beside the importance of HbA1c, prevention of renal complications in adolescents and young adults with type 1 diabetes was associated with certain target levels for non-glycaemic risk factors: blood pressure ≥ 140/80 mmHg, LDL 3.5– < 4.0 mmol/L, BMI ≥ 30 kg/m^2^, HDL < 1.0 mmol/L and total cholesterol ≥ 5.0 mmol/L. Risk was evident already at triglycerides levels ≥ 1.0 mmol/L, which had the strongest impact on risk after glycaemic control and diastolic blood pressure. The current findings are essential for patients and care-givers where treatment for blood pressure, lipids and obesity have potential adverse effects and require major health care resources. Somewhat more tight blood pressure control is indicated in patients with poor glycaemic control being at high risk of nephropathy.

## Supplementary Information

Below is the link to the electronic supplementary material.

Supplementary file1 (DOCX 72 kb)

## Data Availability

Data are not publicly available. Data may be available on request to the Swedish National Diabetes Registry.

## Code availability

N/A.
