# Supplementary material for: Risk factors for nephropathy in persons with type 1 diabetes: a population-based study
Source: Acta Diabetol. 2022 Feb 24;59(6):761–72. doi: 10.1007/s00592-022-01863-6 (PMC9085666; doi:10.1007/s00592-022-01863-6)
Supplement: Supplementary file 1 — Supplementary file1 (DOCX 72 kb) [file 592_2022_1863_MOESM1_ESM.docx]

**Supplement for:**

## Risk Factors for Nephropathy in Persons with Type 1 Diabetes: A Population-Based Study

Shilan Seyed Ahmadi^1,2^, Aldina Pivodic^3,4^, Ann-Marie Svensson^5^, Hans Wedel^6^, Björn Rathsman^7^, Thomas Nyström^8^, Johnny Ludvigsson*^9^, Marcus Lind*^1,2,10^

^1^ Department of Molecular and Clinical Medicine, Institute of Medicine, University of Gothenburg, Gothenburg, Sweden.

^2^ Department of Internal Medicine, Sahlgrenska University Hospital, Gothenburg, Sweden.

^3^ Statistiska Konsultgruppen, Gothenburg, Sweden.

^4^ Department of Clinical Neuroscience, Institute of Neuroscience and Physiology, Sahlgrenska Academy, University of Gothenburg, Gothenburg, Sweden

^5^ Centre of Registers in Region Västra Götaland, Sweden.

^6^ Department of Health Metrics, Sahlgrenska Academy, University of Gothenburg, Sweden.

^7^ Department of Clinical Science and Education, Sachs’ Children and Youth Hospital, Södersjukhuset, Karolinska Institutet, Stockholm, Sweden.

^8^ Department of Clinical Science and Education, Internal Medicine, Södersjukhuset, Karolinska Institutet, Stockholm, Sweden.

^9^ Crown Princess Victoria Children’s Hospital, and Division of Paediatrics, Department of Biomedical and Clinical Sciences, Linköping University, Linköping, Sweden.

^10^ Department of Medicine, NU Hospital Group, Uddevalla, Sweden.

*Shared last author position

Contents

[**Supplemental Table 1.** Patient characteristics for population with recorded albuminuria data at a clinical visit during selected years follow-up from diabetes onset 3](#_Toc71142041)

[**Supplemental Table 2.** Generalized Estimating Equation (GEE) models for the impact of various variables at any time before on albuminuria endpoints. 6](#_Toc71142042)

[**Supplemental Table 3.** Generalized Estimating Equation (GEE) models for the impact of various categories on micro-/macroalbuminuria subgrouping on HbA1c 9](#_Toc71142043)

[**Supplemental Table 4.** Patient characteristics (all patients 13 years of age or later at diabetes diagnosis) 12](#_Toc71142044)

[**Supplemental Table 5.** Generalized Estimating Equation (GEE) models for the impact of various variables at any time before on albuminuria endpoints (all patients 13 years of age or later at diabetes diagnosis) 14](#_Toc71142045)

[**Supplemental Table 6.** Generalized Estimating Equation (GEE) models for the impact of risk factors on nephropathy endpoints (all patients 13 years of age or later at diabetes diagnosis). 17](#_Toc71142046)

## **Supplemental Table 1.** Patient characteristics for population with recorded albuminuria data at a clinical visit during selected years follow-up from diabetes onset

| **Variable** | **Albuminuria 8-10 years (n=6476)** | **Albuminuria 10-12 years (n=4490)** | **Albuminuria 12-14 years (n=2642)** | **Albuminuria 14-16 years (n=1959)** | **Albuminuria 16-20 years (n=1262)** |
| --- | --- | --- | --- | --- | --- |
| **Age at first visit (years)** | 18.1 (7.3) 18.0 (0.6; 34.0) n=6476 | 15.1 (7.1) 13.2 (0.0; 34.0) n=4490 | 10.1 (3.4) 10.1 (0.4; 19.9) n=2642 | 9.34 (3.29) 9.43 (0.66; 19.89) n=1959 | 8.84 (3.20) 8.84 (0.79; 19.71) n=1262 |
| **Sex** |  |  |  |  |  |
| **Male** | 3728 (57.6%) | 2454 (54.7%) | 1341 (50.8%) | 982 (50.1%) | 654 (51.8%) |
| **Female** | 2748 (42.4%) | 2036 (45.3%) | 1301 (49.2%) | 977 (49.9%) | 608 (48.2%) |
| **Onset year** | 2004 (4) 2004 (1993; 2009) n=6476 | 2003 (3) 2004 (1993; 2007) n=4490 | 2002 (2) 2002 (1995; 2005) n=2642 | 2000 (2) 2001 (1995; 2003) n=1959 | 1999 (2) 1999 (1995; 2001) n=1262 |
| **HbA1c AUC 0-10 years from onset** | 62.2 (12.2) 61.0 (29.0; 124.3) n=6476 | 63.9 (11.7) 62.8 (29.1; 129.7) n=4490 | 66.3 (10.5) 65.0 (33.9; 124.7) n=2642 | 67.0 (10.3) 65.5 (41.2; 120.9) n=1959 | 67.0 (10.3) 65.6 (44.0; 109.9) n=1262 |
| **HbA1c AUC (%, NGSP)** | 7.84 (1.11) 7.73 (4.81; 13.53) n=6476 | 8.00 (1.07) 7.90 (4.81; 14.02) n=4490 | 8.22 (0.96) 8.10 (5.25; 13.57) n=2642 | 8.28 (0.94) 8.15 (5.92; 13.21) n=1959 | 8.28 (0.94) 8.15 (6.18; 12.21) n=1262 |
| **HbA1c AUC category** |  |  |  |  |  |
| **<48 mmol/mol** | 661 (10.2%) | 277 (6.2%) | 45 (1.7%) | 21 (1.1%) | 8 (0.6%) |
| **48-52 mmol/mol** | 789 (12.2%) | 452 (10.1%) | 157 (5.9%) | 89 (4.5%) | 62 (4.9%) |
| **53-57 mmol/mol** | 1106 (17.1%) | 702 (15.6%) | 364 (13.8%) | 238 (12.1%) | 163 (12.9%) |
| **58-70 mmol/mol** | 2429 (37.5%) | 1862 (41.5%) | 1268 (48.0%) | 974 (49.7%) | 623 (49.4%) |
| **>70 mmol/mol** | 1491 (23.0%) | 1197 (26.7%) | 808 (30.6%) | 637 (32.5%) | 406 (32.2%) |
| **SBP mean (mmHg)** | 117.5 (9.0) 117.0 (81.3; 175.0) n=6468 | 116.6 (8.5) 116.2 (84.0; 156.7) n=4484 | 115.3 (7.8) 115.0 (90.0; 154.9) n=2640 | 115.2 (7.8) 114.9 (91.1; 148.1) n=1957 | 115.3 (7.7) 115.1 (92.1; 144.6) n=1262 |
| **SBP mean category** |  |  |  |  |  |
| **<110 mmHg** | 1241 (19.2%) | 957 (21.3%) | 644 (24.4%) | 503 (25.7%) | 328 (26.0%) |
| **110-<120 mmHg** | 2806 (43.4%) | 2064 (46.0%) | 1306 (49.5%) | 943 (48.2%) | 595 (47.1%) |
| **120-<130 mmHg** | 1888 (29.2%) | 1156 (25.8%) | 593 (22.5%) | 437 (22.3%) | 288 (22.8%) |
| **130-<140 mmHg** | 452 (7.0%) | 274 (6.1%) | 89 (3.4%) | 68 (3.5%) | 45 (3.6%) |
| **>=140 mmHg** | 81 (1.3%) | 33 (0.7%) | 8 (0.3%) | 6 (0.3%) | 6 (0.5%) |
| **Missing** | 8 | 6 | 2 | 2 | 0 |
| **DBP mean (mmHg)** | 70.5 (6.1) 70.2 (47.1; 105.0) n=6468 | 69.8 (5.7) 69.6 (45.0; 95.0) n=4484 | 68.7 (4.9) 68.5 (52.3; 86.3) n=2640 | 68.6 (5.0) 68.5 (51.8; 88.4) n=1957 | 68.8 (5.0) 68.6 (55.4; 88.6) n=1262 |
| **DBP mean category** |  |  |  |  |  |
| **<60 mmHg** | 195 (3.0%) | 142 (3.2%) | 77 (2.9%) | 74 (3.8%) | 52 (4.1%) |
| **60-<70 mmHg** | 2848 (44.0%) | 2188 (48.8%) | 1542 (58.4%) | 1120 (57.2%) | 723 (57.3%) |
| **70-<80 mmHg** | 2977 (46.0%) | 1949 (43.5%) | 983 (37.2%) | 729 (37.3%) | 464 (36.8%) |
| **80-<85 mmHg** | 353 (5.5%) | 167 (3.7%) | 35 (1.3%) | 30 (1.5%) | 19 (1.5%) |
| **>=85 mmHg** | 95 (1.5%) | 38 (0.8%) | 3 (0.1%) | 4 (0.2%) | 4 (0.3%) |
| **Missing** | 8 | 6 | 2 | 2 | 0 |
| **BMI mean (kg/m^2)** | 23.5 (4.1) 23.0 (14.3; 48.3) n=6446 | 22.9 (3.9) 22.4 (13.2; 48.4) n=4477 | 21.8 (3.4) 21.2 (14.8; 39.7) n=2642 | 21.7 (3.3) 21.2 (14.2; 39.6) n=1958 | 21.7 (3.3) 21.2 (14.3; 38.5) n=1261 |
| **BMI mean category** |  |  |  |  |  |
| **<18.5 kg/m^2** | 502 (7.8%) | 451 (10.1%) | 380 (14.4%) | 275 (14.0%) | 170 (13.5%) |
| **18.5-<25 kg/m^2** | 3990 (61.9%) | 2891 (64.6%) | 1854 (70.2%) | 1401 (71.6%) | 920 (73.0%) |
| **25-<30 kg/m^2** | 1555 (24.1%) | 922 (20.6%) | 340 (12.9%) | 234 (12.0%) | 145 (11.5%) |
| **30-<35 kg/m^2** | 317 (4.9%) | 175 (3.9%) | 60 (2.3%) | 41 (2.1%) | 20 (1.6%) |
| **>=35 kg/m^2** | 82 (1.3%) | 38 (0.8%) | 8 (0.3%) | 7 (0.4%) | 6 (0.5%) |
| **Missing** | 30 | 13 | 0 | 1 | 1 |
| **HDL mean (mmol/L)** | 1.51 (0.39) 1.47 (0.53; 5.90) n=5904 | 1.52 (0.40) 1.48 (0.53; 5.00) n=4126 | 1.51 (0.39) 1.47 (0.40; 4.40) n=2431 | 1.51 (0.37) 1.47 (0.58; 3.90) n=1847 | 1.51 (0.35) 1.47 (0.73; 3.90) n=1220 |
| **HDL mean category** |  |  |  |  |  |
| **<1.0 mmol/L** | 275 (4.7%) | 198 (4.8%) | 110 (4.5%) | 87 (4.7%) | 44 (3.6%) |
| **1.0-<1.5 mmol/L** | 2818 (47.7%) | 1954 (47.4%) | 1160 (47.7%) | 881 (47.7%) | 599 (49.1%) |
| **1.5-<2.0 mmol/L** | 2200 (37.3%) | 1527 (37.0%) | 912 (37.5%) | 704 (38.1%) | 472 (38.7%) |
| **>=2.0 mmol/L** | 611 (10.3%) | 447 (10.8%) | 249 (10.2%) | 175 (9.5%) | 105 (8.6%) |
| **Missing** | 572 | 364 | 211 | 112 | 42 |
| **LDL mean (mmol/L)** | 2.55 (0.69) 2.48 (0.73; 9.04) n=5886 | 2.53 (0.71) 2.46 (-0.36; 8.94) n=4131 | 2.52 (0.70) 2.45 (0.72; 7.09) n=2428 | 2.53 (0.69) 2.46 (0.42; 5.70) n=1859 | 2.52 (0.65) 2.45 (0.77; 5.17) n=1239 |
| **LDL mean category** |  |  |  |  |  |
| **<2.0 mmol/L** | 1204 (20.5%) | 907 (22.0%) | 538 (22.2%) | 411 (22.1%) | 255 (20.6%) |
| **2.0-<2.5 mmol/L** | 1794 (30.5%) | 1257 (30.4%) | 746 (30.7%) | 556 (29.9%) | 406 (32.8%) |
| **2.5-<3.0 mmol/L** | 1507 (25.6%) | 1034 (25.0%) | 595 (24.5%) | 476 (25.6%) | 318 (25.7%) |
| **3.0-<3.5 mmol/L** | 855 (14.5%) | 571 (13.8%) | 332 (13.7%) | 249 (13.4%) | 151 (12.2%) |
| **3.5-<4.0 mmol/L** | 358 (6.1%) | 248 (6.0%) | 144 (5.9%) | 113 (6.1%) | 78 (6.3%) |
| **>=4.0 mmol/L** | 168 (2.9%) | 114 (2.8%) | 73 (3.0%) | 54 (2.9%) | 31 (2.5%) |
| **Missing** | 590 | 359 | 214 | 100 | 23 |
| **Cholesterol mean (mmol/L)** | 4.51 (0.80) 4.45 (2.10; 11.00) n=6002 | 4.49 (0.82) 4.41 (1.80; 11.00) n=4200 | 4.49 (0.81) 4.41 (1.60; 9.70) n=2481 | 4.48 (0.79) 4.40 (2.20; 8.63) n=1866 | 4.48 (0.76) 4.42 (2.20; 8.20) n=1231 |
| **Cholesterol mean category** |  |  |  |  |  |
| **<4.0 mmol/L** | 1526 (25.4%) | 1113 (26.5%) | 670 (27.0%) | 493 (26.4%) | 304 (24.7%) |
| **4.0-<5.0 mmol/L** | 2973 (49.5%) | 2074 (49.4%) | 1207 (48.6%) | 955 (51.2%) | 645 (52.4%) |
| **5.0-<6.0 mmol/L** | 1250 (20.8%) | 835 (19.9%) | 497 (20.0%) | 342 (18.3%) | 234 (19.0%) |
| **>=6.0 mmol/L** | 253 (4.2%) | 178 (4.2%) | 107 (4.3%) | 76 (4.1%) | 48 (3.9%) |
| **Missing** | 474 | 290 | 161 | 93 | 31 |
| **Triglycerides mean (mmol/L)** | 1.07 (0.72) 0.90 (0.20; 18.06) n=5788 | 1.07 (0.66) 0.90 (0.10; 11.50) n=4034 | 1.09 (0.61) 0.93 (0.20; 6.52) n=2336 | 1.09 (0.61) 0.93 (0.20; 9.33) n=1768 | 1.09 (0.56) 0.94 (0.36; 5.31) n=1171 |
| **Triglycerides mean category** |  |  |  |  |  |
| **<0.5 mmol/L** | 263 (4.5%) | 175 (4.3%) | 83 (3.6%) | 61 (3.5%) | 29 (2.5%) |
| **0.5-<1.0 mmol/L** | 3127 (54.0%) | 2182 (54.1%) | 1215 (52.0%) | 930 (52.6%) | 612 (52.3%) |
| **1.0-<1.5 mmol/L** | 1505 (26.0%) | 1060 (26.3%) | 642 (27.5%) | 496 (28.1%) | 347 (29.6%) |
| **1.5-2.0 mmol/L** | 491 (8.5%) | 349 (8.7%) | 213 (9.1%) | 161 (9.1%) | 103 (8.8%) |
| **>=2.0 mmol/L** | 402 (6.9%) | 268 (6.6%) | 183 (7.8%) | 120 (6.8%) | 80 (6.8%) |
| **Missing** | 688 | 456 | 306 | 191 | 91 |
| **Smoking at any time before** |  |  |  |  |  |
| **No** | 4715 (79.3%) | 3295 (79.6%) | 1901 (78.8%) | 1466 (78.9%) | 985 (79.0%) |
| **Yes** | 1233 (20.7%) | 842 (20.4%) | 511 (21.2%) | 391 (21.1%) | 262 (21.0%) |
| **Missing** | 528 | 353 | 230 | 102 | 15 |
| For categorical variables n (%) is presented. For continuous variables Mean (SD) / Median (Min; Max) / n= is presented. | | | | | |

## **Supplemental Table 2.** Generalized Estimating Equation (GEE) models for the impact of various variables at any time before on albuminuria endpoints.

|  | | | | **Adjusted for age and sex** | | **Adjusted for age, sex and HbA1c mean** | |
| --- | --- | --- | --- | --- | --- | --- | --- |
| **Variable** | **Category** | **n (%) (95% CI) events** | **n (%) (95% CI) persons with events** | **OR (95% CI)** | **p-value** | **OR (95% CI)** | **p-value** |
| **Microalbuminuria/Macroalbuminuria vs None** | | | | | | | |
| **SBP** | **<110 mmHg** | 193 (5.3%) (4.6% - 6.0%) | 153 (6.8%) (5.8% - 7.9%) | 0.93 (0.76-1.13) | 0.45 | 0.97 (0.80-1.18) | 0.78 |
|  | **110-<120 mmHg** | 455 (5.9%) (5.4% - 6.4%) | 333 (7.5%) (6.7% - 8.3%) | 1.00 |  | 1.00 |  |
|  | **120-<130 mmHg** | 276 (6.3%) (5.6% - 7.1%) | 202 (7.6%) (6.6% - 8.7%) | 1.06 (0.88-1.28) | 0.55 | 1.07 (0.88-1.30) | 0.48 |
|  | **130-<140 mmHg** | 76 (8.2%) (6.5% - 10.1%) | 57 (9.4%) (7.3% - 12.1%) | 1.32 (0.96-1.81) | 0.089 | 1.29 (0.95-1.75) | 0.11 |
|  | **>=140 mmHg** | 26 (19.4%) (13.1% - 27.1%) | 23 (23.0%) (15.9% - 33.3%) | 3.39 (2.10-5.48) | <.0001 | 3.50 (2.17-5.63) | <.0001 |
| **DBP** | **<60 mmHg** | 26 (4.8%) (3.2% - 7.0%) | 21 (5.5%) (3.6% - 8.5%) | 0.92 (0.59-1.44) | 0.71 | 1.03 (0.64-1.67) | 0.89 |
|  | **60-<70 mmHg** | 417 (5.0%) (4.5% - 5.4%) | 308 (6.4%) (5.8% - 7.2%) | 1.00 |  | 1.00 |  |
|  | **70-<80 mmHg** | 480 (6.8%) (6.2% - 7.4%) | 343 (8.1%) (7.3% - 9.0%) | 1.23 (1.04-1.46) | 0.018 | 1.14 (0.96-1.34) | 0.13 |
|  | **80-<85 mmHg** | 67 (11.1%) (8.7% - 13.9%) | 53 (12.0%) (9.3% - 15.6%) | 2.13 (1.55-2.94) | <.0001 | 1.81 (1.33-2.45) | 0.0001 |
|  | **>=85 mmHg** | 36 (25.0%) (18.2% - 32.9%) | 31 (27.4%) (20.1% - 37.3%) | 5.38 (3.52-8.22) | <.0001 | 4.60 (2.99-7.07) | <.0001 |
| **BMI** | **<18.5 kg/m^2** | 119 (6.7%) (5.6% - 8.0%) | 85 (7.3%) (6.0% - 9.1%) | 1.19 (0.87-1.61) | 0.27 | 1.31 (0.99-1.73) | 0.062 |
|  | **18.5-<25 kg/m^2** | 607 (5.5%) (5.1% - 5.9%) | 438 (7.3%) (6.7% - 8.0%) | 1.00 |  | 1.00 |  |
|  | **25-<30 kg/m^2** | 199 (6.2%) (5.4% - 7.1%) | 152 (7.8%) (6.7% - 9.1%) | 1.00 (0.82-1.22) | 1.00 | 0.96 (0.79-1.17) | 0.68 |
|  | **30-<35 kg/m^2** | 63 (10.3%) (8.0% - 13.0%) | 49 (12.5%) (9.6% - 16.4%) | 1.74 (1.25-2.41) | 0.0009 | 1.43 (1.03-2.00) | 0.033 |
|  | **>=35 kg/m^2** | 34 (24.1%) (17.3% - 32.0%) | 24 (24.2%) (16.9% - 34.7%) | 3.91 (2.49-6.14) | <.0001 | 2.79 (1.74-4.47) | <.0001 |
| **LDL** | **<2.0 mmol/L** | 166 (5.0%) (4.3% - 5.8%) | 130 (6.4%) (5.4% - 7.6%) | 0.98 (0.78-1.25) | 0.90 | 1.00 (0.79-1.26) | 0.97 |
|  | **2.0-<2.5 mmol/L** | 257 (5.4%) (4.8% - 6.1%) | 196 (6.6%) (5.8% - 7.6%) | 1.00 |  | 1.00 |  |
|  | **2.5-<3.0 mmol/L** | 246 (6.3%) (5.5% - 7.1%) | 186 (7.4%) (6.5% - 8.6%) | 1.15 (0.92-1.43) | 0.21 | 1.08 (0.88-1.34) | 0.46 |
|  | **3.0-<3.5 mmol/L** | 156 (7.2%) (6.2% - 8.4%) | 129 (9.1%) (7.7% - 10.8%) | 1.36 (1.07-1.72) | 0.012 | 1.17 (0.92-1.48) | 0.21 |
|  | **3.5-<4.0 mmol/L** | 83 (8.8%) (7.1% - 10.8%) | 69 (10.9%) (8.7% - 13.8%) | 1.72 (1.30-2.26) | 0.0001 | 1.39 (1.05-1.83) | 0.020 |
|  | **>=4.0 mmol/L** | 49 (11.1%) (8.4% - 14.5%) | 35 (12.3%) (9.0% - 17.1%) | 1.92 (1.25-2.93) | 0.0027 | 1.35 (0.88-2.08) | 0.17 |
| **HDL** | **<1.0 mmol/L** | 68 (9.5%) (7.5% - 11.9%) | 52 (11.8%) (9.2% - 15.5%) | 1.73 (1.25-2.40) | 0.0009 | 1.48 (1.08-2.02) | 0.013 |
|  | **1.0-<1.5 mmol/L** | 463 (6.2%) (5.7% - 6.8%) | 342 (8.0%) (7.3% - 8.9%) | 1.00 |  | 1.00 |  |
|  | **1.5-<2.0 mmol/L** | 343 (5.9%) (5.3% - 6.5%) | 249 (7.2%) (6.4% - 8.1%) | 0.93 (0.78-1.10) | 0.38 | 0.97 (0.82-1.15) | 0.76 |
|  | **>=2.0 mmol/L** | 85 (5.4%) (4.3% - 6.6%) | 66 (6.7%) (5.3% - 8.5%) | 0.82 (0.62-1.07) | 0.15 | 0.93 (0.71-1.22) | 0.60 |
| **Triglycerides** | **<0.5 mmol/L** | 33 (5.4%) (3.7% - 7.5%) | 27 (6.5%) (4.5% - 9.6%) | 1.10 (0.74-1.62) | 0.65 | 1.22 (0.82-1.80) | 0.32 |
|  | **0.5-<1.0 mmol/L** | 384 (4.8%) (4.3% - 5.2%) | 284 (6.0%) (5.3% - 6.7%) | 1.00 |  | 1.00 |  |
|  | **1.0-<1.5 mmol/L** | 256 (6.3%) (5.6% - 7.1%) | 200 (8.0%) (7.0% - 9.2%) | 1.41 (1.18-1.70) | 0.0002 | 1.21 (1.01-1.46) | 0.039 |
|  | **1.5-2.0 mmol/L** | 109 (8.3%) (6.8% - 9.9%) | 84 (9.5%) (7.7% - 11.7%) | 1.78 (1.37-2.31) | <.0001 | 1.37 (1.05-1.78) | 0.020 |
|  | **>=2.0 mmol/L** | 148 (14.1%) (12.0% - 16.3%) | 110 (17.2%) (14.4% - 20.4%) | 3.35 (2.61-4.29) | <.0001 | 2.17 (1.67-2.81) | <.0001 |
| **Cholesterol** | **<4.0 mmol/L** | 198 (4.8%) (4.2% - 5.5%) | 151 (6.2%) (5.3% - 7.2%) | 0.93 (0.75-1.15) | 0.51 | 0.98 (0.79-1.21) | 0.86 |
|  | **4.0-<5.0 mmol/L** | 425 (5.4%) (4.9% - 5.9%) | 318 (6.8%) (6.1% - 7.6%) | 1.00 |  | 1.00 |  |
|  | **5.0-<6.0 mmol/L** | 268 (8.5%) (7.5% - 9.5%) | 203 (10.5%) (9.2% - 12.0%) | 1.71 (1.41-2.06) | <.0001 | 1.51 (1.25-1.81) | <.0001 |
|  | **>=6.0 mmol/L** | 77 (11.6%) (9.3% - 14.3%) | 53 (13.2%) (10.2% - 17.1%) | 2.38 (1.73-3.29) | <.0001 | 1.79 (1.30-2.45) | 0.0003 |
| **Smoking** | **No** | 711 (5.8%) (5.3% - 6.2%) | 505 (7.5%) (6.9% - 8.2%) | 1.00 |  | 1.00 |  |
|  | **Yes** | 264 (8.2%) (7.2% - 9.1%) | 196 (10.5%) (9.2% - 12.1%) | 1.42 (1.19-1.69) | <.0001 | 1.05 (0.88-1.26) | 0.58 |
| **Macroalbuminuria vs None/Microalbuminuria** | | | | | | | |
| **SBP** | **<110 mmHg** | 32 (0.9%) (0.6% - 1.2%) | 28 (1.2%) (0.9% - 1.8%) | 0.94 (0.57-1.52) | 0.79 | 1.00 (0.62-1.59) | 0.99 |
|  | **110-<120 mmHg** | 71 (0.9%) (0.7% - 1.2%) | 59 (1.3%) (1.0% - 1.7%) | 1.00 |  | 1.00 |  |
|  | **120-<130 mmHg** | 37 (0.8%) (0.6% - 1.2%) | 30 (1.1%) (0.8% - 1.6%) | 1.02 (0.64-1.61) | 0.95 | 0.91 (0.58-1.44) | 0.69 |
|  | **130-<140 mmHg** | 12 (1.3%) (0.7% - 2.2%) | 11 (1.8%) (1.0% - 3.4%) | 1.72 (0.85-3.51) | 0.13 | 1.49 (0.79-2.81) | 0.22 |
|  | **>=140 mmHg** | 11 (8.2%) (4.2% - 14.2%) | 10 (10.0%) (5.6% - 18.7%) | 5.49 (1.45-20.80) | 0.012 | 6.81 (3.16-14.68) | <.0001 |
| **DBP** | **<60 mmHg** | 4 (0.7%) (0.2% - 1.9%) | 4 (1.0%) (0.4% - 3.0%) | 1.12 (0.46-2.74) | 0.80 | 1.18 (0.46-3.06) | 0.73 |
|  | **60-<70 mmHg** | 71 (0.8%) (0.7% - 1.1%) | 59 (1.2%) (1.0% - 1.6%) | 1.00 |  | 1.00 |  |
|  | **70-<80 mmHg** | 63 (0.9%) (0.7% - 1.1%) | 50 (1.2%) (0.9% - 1.6%) | 0.85 (0.57-1.26) | 0.42 | 0.80 (0.53-1.22) | 0.30 |
|  | **80-<85 mmHg** | 10 (1.7%) (0.8% - 3.0%) | 9 (2.0%) (1.1% - 4.1%) | 1.41 (0.70-2.82) | 0.33 | 1.20 (0.58-2.49) | 0.63 |
|  | **>=85 mmHg** | 15 (10.4%) (5.9% - 16.6%) | 14 (12.4%) (7.6% - 20.8%) | 6.33 (2.34-17.12) | 0.0003 | 7.35 (3.69-14.62) | <.0001 |
| **BMI** | **<18.5 kg/m^2** | 15 (0.8%) (0.5% - 1.4%) | 13 (1.1%) (0.7% - 2.0%) | 1.27 (0.69-2.36) | 0.44 | 1.43 (0.77-2.64) | 0.26 |
|  | **18.5-<25 kg/m^2** | 101 (0.9%) (0.7% - 1.1%) | 77 (1.3%) (1.0% - 1.6%) | 1.00 |  | 1.00 |  |
|  | **25-<30 kg/m^2** | 30 (0.9%) (0.6% - 1.3%) | 28 (1.4%) (1.0% - 2.1%) | 0.98 (0.64-1.50) | 0.94 | 0.90 (0.58-1.39) | 0.64 |
|  | **30-<35 kg/m^2** | 11 (1.8%) (0.9% - 3.2%) | 11 (2.8%) (1.6% - 5.3%) | 1.66 (0.84-3.28) | 0.14 | 1.18 (0.54-2.61) | 0.68 |
|  | **>=35 kg/m^2** | 5 (3.5%) (1.2% - 8.1%) | 4 (4.0%) (1.6% - 11.3%) | 2.00 (0.70-5.72) | 0.20 | 1.62 (0.54-4.87) | 0.39 |
| **LDL** | **<2.0 mmol/L** | 25 (0.8%) (0.5% - 1.1%) | 20 (1.0%) (0.6% - 1.6%) | 1.48 (0.80-2.72) | 0.21 | 1.20 (0.70-2.07) | 0.50 |
|  | **2.0-<2.5 mmol/L** | 33 (0.7%) (0.5% - 1.0%) | 29 (1.0%) (0.7% - 1.4%) | 1.00 |  | 1.00 |  |
|  | **2.5-<3.0 mmol/L** | 36 (0.9%) (0.6% - 1.3%) | 34 (1.4%) (1.0% - 1.9%) | 1.54 (0.80-2.97) | 0.19 | 1.32 (0.81-2.13) | 0.26 |
|  | **3.0-<3.5 mmol/L** | 22 (1.0%) (0.6% - 1.5%) | 20 (1.4%) (0.9% - 2.3%) | 1.48 (0.64-3.44) | 0.36 | 0.96 (0.51-1.81) | 0.91 |
|  | **3.5-<4.0 mmol/L** | 16 (1.7%) (1.0% - 2.7%) | 13 (2.1%) (1.2% - 3.7%) | 2.32 (1.21-4.45) | 0.011 | 1.85 (0.99-3.47) | 0.054 |
|  | **>=4.0 mmol/L** | 12 (2.7%) (1.4% - 4.7%) | 7 (2.5%) (1.2% - 5.5%) | 3.09 (1.25-7.63) | 0.015 | 2.03 (0.87-4.77) | 0.10 |
| **HDL** | **<1.0 mmol/L** | 12 (1.7%) (0.9% - 2.9%) | 11 (2.5%) (1.4% - 4.7%) | 2.21 (1.18-4.15) | 0.013 | 1.78 (0.92-3.42) | 0.086 |
|  | **1.0-<1.5 mmol/L** | 66 (0.9%) (0.7% - 1.1%) | 54 (1.3%) (1.0% - 1.7%) | 1.00 |  | 1.00 |  |
|  | **1.5-<2.0 mmol/L** | 52 (0.9%) (0.7% - 1.2%) | 43 (1.2%) (0.9% - 1.7%) | 0.88 (0.58-1.33) | 0.54 | 0.97 (0.65-1.47) | 0.90 |
|  | **>=2.0 mmol/L** | 18 (1.1%) (0.7% - 1.8%) | 13 (1.3%) (0.8% - 2.4%) | 1.00 (0.53-1.88) | 0.99 | 1.22 (0.64-2.32) | 0.54 |
| **Triglycerides** | **<0.5 mmol/L** | 3 (0.5%) (0.1% - 1.4%) | 2 (0.5%) (0.1% - 2.1%) | 0.91 (0.26-3.22) | 0.88 | 0.99 (0.27-3.58) | 0.98 |
|  | **0.5-<1.0 mmol/L** | 45 (0.6%) (0.4% - 0.7%) | 37 (0.8%) (0.6% - 1.1%) | 1.00 |  | 1.00 |  |
|  | **1.0-<1.5 mmol/L** | 36 (0.9%) (0.6% - 1.2%) | 29 (1.2%) (0.8% - 1.7%) | 1.70 (1.04-2.79) | 0.034 | 1.44 (0.87-2.38) | 0.15 |
|  | **1.5-2.0 mmol/L** | 16 (1.2%) (0.7% - 2.0%) | 15 (1.7%) (1.0% - 2.9%) | 2.05 (0.98-4.31) | 0.057 | 1.58 (0.75-3.32) | 0.23 |
|  | **>=2.0 mmol/L** | 37 (3.5%) (2.5% - 4.8%) | 29 (4.5%) (3.2% - 6.6%) | 7.20 (4.33-11.95) | <.0001 | 4.67 (2.72-8.02) | <.0001 |
| **Cholesterol** | **<4.0 mmol/L** | 24 (0.6%) (0.4% - 0.9%) | 20 (0.8%) (0.5% - 1.3%) | 0.89 (0.52-1.52) | 0.67 | 0.94 (0.55-1.59) | 0.81 |
|  | **4.0-<5.0 mmol/L** | 59 (0.8%) (0.6% - 1.0%) | 52 (1.1%) (0.9% - 1.5%) | 1.00 |  | 1.00 |  |
|  | **5.0-<6.0 mmol/L** | 41 (1.3%) (0.9% - 1.8%) | 37 (1.9%) (1.4% - 2.7%) | 1.54 (1.00-2.39) | 0.052 | 1.32 (0.85-2.05) | 0.21 |
|  | **>=6.0 mmol/L** | 23 (3.5%) (2.2% - 5.2%) | 16 (4.0%) (2.5% - 6.7%) | 4.30 (2.40-7.71) | <.0001 | 3.16 (1.80-5.56) | <.0001 |
| **Smoking** | **No** | 104 (0.8%) (0.7% - 1.0%) | 83 (1.2%) (1.0% - 1.5%) | 1.00 |  | 1.00 |  |
|  | **Yes** | 52 (1.6%) (1.2% - 2.1%) | 43 (2.3%) (1.7% - 3.2%) | 1.81 (1.23-2.67) | 0.0027 | 1.32 (0.88-1.98) | 0.17 |

## **Supplemental Table 3.** Generalized Estimating Equation (GEE) models for the impact of various categories on micro-/macroalbuminuria subgrouping on HbA1c

|  | | | | **Adjusted for age and sex** | | **Adjusted for age, sex and HbA1c mean** | |
| --- | --- | --- | --- | --- | --- | --- | --- |
| **Variable** | **category** | **n (%) (95% CI) events** | **n (%) (95% CI) persons with events** | **OR (95% CI)** | **p-value** | **OR (95% CI)** | **p-value** |
| **Microalbuminuria/Macroalbuminuria vs None (<=65 mmol/mol)** | | | | | | | |
| **SBP** | **<110 mmHg** | 94 (4.2%) (3.4% - 5.1%) | 76 (5.2%) (4.2% - 6.5%) | 1.10 (0.83-1.47) | 0.50 | 1.11 (0.84-1.49) | 0.46 |
|  | **110-<120 mmHg** | 165 (3.8%) (3.3% - 4.4%) | 129 (4.8%) (4.1% - 5.7%) | 1.00 |  | 1.00 |  |
|  | **120-<130 mmHg** | 109 (4.5%) (3.7% - 5.4%) | 85 (5.4%) (4.4% - 6.7%) | 1.07 (0.79-1.45) | 0.67 | 1.07 (0.79-1.45) | 0.67 |
|  | **130-<140 mmHg** | 28 (5.8%) (3.9% - 8.3%) | 19 (5.6%) (3.6% - 8.9%) | 1.44 (0.84-2.49) | 0.18 | 1.44 (0.84-2.49) | 0.19 |
|  | **>=140 mmHg** | 8 (10.3%) (4.5% - 19.2%) | 8 (13.3%) (7.0% - 26.2%) | 3.39 (1.63-7.04) | 0.0011 | 3.39 (1.63-7.02) | 0.0010 |
| **DBP** | **<60 mmHg** | 13 (3.4%) (1.8% - 5.7%) | 11 (3.8%) (2.2% - 7.2%) | 0.76 (0.39-1.47) | 0.41 | 0.77 (0.40-1.48) | 0.43 |
|  | **60-<70 mmHg** | 199 (4.0%) (3.4% - 4.5%) | 147 (4.9%) (4.1% - 5.7%) | 1.00 |  | 1.00 |  |
|  | **70-<80 mmHg** | 154 (4.1%) (3.5% - 4.8%) | 121 (5.0%) (4.2% - 6.0%) | 0.95 (0.72-1.24) | 0.70 | 0.95 (0.72-1.24) | 0.69 |
|  | **80-<85 mmHg** | 24 (7.8%) (5.1% - 11.4%) | 19 (8.3%) (5.4% - 13.1%) | 1.75 (1.06-2.90) | 0.030 | 1.69 (1.02-2.82) | 0.043 |
|  | **>=85 mmHg** | 14 (20.9%) (11.9% - 32.6%) | 13 (23.2%) (14.1% - 37.8%) | 5.31 (2.70-10.45) | <.0001 | 5.14 (2.60-10.13) | <.0001 |
| **BMI** | **<18.5 kg/m^2** | 50 (4.4%) (3.3% - 5.7%) | 38 (4.8%) (3.5% - 6.6%) | 1.15 (0.74-1.78) | 0.54 | 1.17 (0.75-1.82) | 0.49 |
|  | **18.5-<25 kg/m^2** | 238 (3.8%) (3.3% - 4.2%) | 186 (5.0%) (4.4% - 5.8%) | 1.00 |  | 1.00 |  |
|  | **25-<30 kg/m^2** | 83 (4.8%) (3.8% - 5.9%) | 64 (5.6%) (4.4% - 7.2%) | 1.10 (0.80-1.50) | 0.56 | 1.07 (0.78-1.46) | 0.67 |
|  | **30-<35 kg/m^2** | 20 (7.3%) (4.5% - 11.0%) | 16 (8.2%) (5.1% - 13.5%) | 1.84 (1.06-3.19) | 0.030 | 1.73 (1.00-3.01) | 0.051 |
|  | **>=35 kg/m^2** | 10 (24.4%) (12.4% - 40.3%) | 7 (21.9%) (11.1% - 42.3%) | 5.19 (2.18-12.37) | 0.0002 | 4.91 (2.08-11.63) | 0.0003 |
| **LDL** | **<2.0 mmol/L** | 87 (4.2%) (3.4% - 5.2%) | 67 (5.1%) (4.1% - 6.5%) | 1.07 (0.77-1.49) | 0.69 | 1.08 (0.77-1.51) | 0.65 |
|  | **2.0-<2.5 mmol/L** | 112 (3.9%) (3.2% - 4.7%) | 86 (4.7%) (3.8% - 5.8%) | 1.00 |  | 1.00 |  |
|  | **2.5-<3.0 mmol/L** | 95 (4.4%) (3.5% - 5.3%) | 73 (5.1%) (4.1% - 6.4%) | 1.01 (0.73-1.39) | 0.95 | 1.00 (0.73-1.38) | 1.00 |
|  | **3.0-<3.5 mmol/L** | 57 (5.3%) (4.1% - 6.8%) | 49 (6.6%) (5.0% - 8.7%) | 1.30 (0.89-1.88) | 0.17 | 1.27 (0.87-1.84) | 0.21 |
|  | **3.5-<4.0 mmol/L** | 22 (5.5%) (3.5% - 8.2%) | 19 (6.7%) (4.4% - 10.7%) | 1.28 (0.79-2.08) | 0.31 | 1.25 (0.78-2.01) | 0.36 |
|  | **>=4.0 mmol/L** | 6 (3.7%) (1.4% - 7.8%) | 4 (3.6%) (1.5% - 10.0%) | 0.91 (0.37-2.24) | 0.84 | 0.88 (0.36-2.16) | 0.78 |
| **HDL** | **<1.0 mmol/L** | 14 (4.9%) (2.7% - 8.2%) | 10 (5.3%) (2.9% - 10.2%) | 1.31 (0.74-2.29) | 0.35 | 1.31 (0.74-2.30) | 0.36 |
|  | **1.0-<1.5 mmol/L** | 175 (4.4%) (3.8% - 5.1%) | 137 (5.6%) (4.7% - 6.6%) | 1.00 |  | 1.00 |  |
|  | **1.5-<2.0 mmol/L** | 142 (4.1%) (3.5% - 4.8%) | 104 (4.8%) (4.0% - 5.9%) | 0.86 (0.66-1.13) | 0.29 | 0.87 (0.67-1.14) | 0.32 |
|  | **>=2.0 mmol/L** | 42 (4.1%) (3.0% - 5.5%) | 33 (4.9%) (3.5% - 7.0%) | 0.85 (0.57-1.27) | 0.43 | 0.86 (0.57-1.28) | 0.45 |
| **Triglycerides** | **<0.5 mmol/L** | 15 (3.3%) (1.8% - 5.3%) | 14 (4.5%) (2.7% - 7.8%) | 0.81 (0.45-1.46) | 0.48 | 0.83 (0.46-1.50) | 0.53 |
|  | **0.5-<1.0 mmol/L** | 204 (3.9%) (3.4% - 4.4%) | 154 (4.8%) (4.1% - 5.6%) | 1.00 |  | 1.00 |  |
|  | **1.0-<1.5 mmol/L** | 102 (5.0%) (4.1% - 6.1%) | 82 (6.1%) (5.0% - 7.6%) | 1.49 (1.16-1.92) | 0.0022 | 1.46 (1.12-1.88) | 0.0043 |
|  | **1.5-2.0 mmol/L** | 25 (5.0%) (3.2% - 7.2%) | 19 (5.2%) (3.4% - 8.4%) | 1.25 (0.74-2.11) | 0.40 | 1.24 (0.74-2.10) | 0.42 |
|  | **>=2.0 mmol/L** | 19 (6.6%) (4.0% - 10.0%) | 17 (8.7%) (5.5% - 14.1%) | 2.29 (1.42-3.67) | 0.0006 | 2.18 (1.36-3.50) | 0.0013 |
| **Cholesterol** | **<4.0 mmol/L** | 114 (4.4%) (3.7% - 5.3%) | 82 (5.1%) (4.1% - 6.3%) | 1.20 (0.89-1.62) | 0.23 | 1.23 (0.91-1.65) | 0.18 |
|  | **4.0-<5.0 mmol/L** | 168 (3.7%) (3.2% - 4.3%) | 135 (4.7%) (4.0% - 5.6%) | 1.00 |  | 1.00 |  |
|  | **5.0-<6.0 mmol/L** | 82 (5.2%) (4.2% - 6.5%) | 62 (6.1%) (4.8% - 7.8%) | 1.45 (1.04-2.01) | 0.027 | 1.43 (1.03-1.98) | 0.033 |
|  | **>=6.0 mmol/L** | 12 (4.9%) (2.6% - 8.5%) | 8 (5.1%) (2.6% - 10.5%) | 1.37 (0.63-2.97) | 0.42 | 1.33 (0.62-2.88) | 0.46 |
| **Smoking** | **No** | 320 (4.3%) (3.8% - 4.7%) | 232 (5.3%) (4.7% - 6.1%) | 1.00 |  | 1.00 |  |
|  | **Yes** | 56 (4.8%) (3.6% - 6.2%) | 49 (6.5%) (5.0% - 8.7%) | 1.16 (0.84-1.61) | 0.36 | 1.11 (0.80-1.53) | 0.53 |
| **Microalbuminuria/Macroalbuminuria vs None (>65 mmol/mol)** | | | | | | | |
| **SBP** | **<110 mmHg** | 99 (7.0%) (5.7% - 8.4%) | 77 (9.2%) (7.4% - 11.5%) | 0.89 (0.68-1.17) | 0.40 | 0.86 (0.66-1.12) | 0.26 |
|  | **110-<120 mmHg** | 290 (8.5%) (7.6% - 9.5%) | 204 (11.0%) (9.6% - 12.5%) | 1.00 |  | 1.00 |  |
|  | **120-<130 mmHg** | 167 (8.6%) (7.4% - 10.0%) | 118 (10.4%) (8.8% - 12.4%) | 0.96 (0.75-1.24) | 0.78 | 1.02 (0.79-1.30) | 0.89 |
|  | **130-<140 mmHg** | 48 (10.7%) (8.0% - 14.0%) | 38 (13.7%) (10.2% - 18.7%) | 1.20 (0.82-1.78) | 0.35 | 1.30 (0.89-1.90) | 0.17 |
|  | **>=140 mmHg** | 18 (32.1%) (20.3% - 46.0%) | 15 (36.6%) (23.6% - 54.4%) | 3.04 (1.56-5.93) | 0.0011 | 3.45 (1.78-6.69) | 0.0002 |
| **DBP** | **<60 mmHg** | 13 (8.4%) (4.5% - 13.9%) | 10 (10.2%) (5.7% - 19.0%) | 1.37 (0.71-2.64) | 0.35 | 1.44 (0.72-2.86) | 0.30 |
|  | **60-<70 mmHg** | 218 (6.4%) (5.6% - 7.3%) | 161 (8.7%) (7.5% - 10.1%) | 1.00 |  | 1.00 |  |
|  | **70-<80 mmHg** | 326 (9.8%) (8.8% - 10.8%) | 223 (11.9%) (10.5% - 13.5%) | 1.30 (1.04-1.64) | 0.024 | 1.28 (1.02-1.60) | 0.030 |
|  | **80-<85 mmHg** | 43 (14.5%) (10.7% - 19.0%) | 34 (15.8%) (11.6% - 21.8%) | 1.82 (1.16-2.85) | 0.0094 | 1.88 (1.22-2.91) | 0.0046 |
|  | **>=85 mmHg** | 22 (28.6%) (18.8% - 40.0%) | 18 (30.5%) (20.3% - 45.0%) | 4.31 (2.46-7.56) | <.0001 | 4.72 (2.68-8.33) | <.0001 |
| **BMI** | **<18.5 kg/m^2** | 69 (10.8%) (8.5% - 13.5%) | 47 (12.3%) (9.4% - 16.3%) | 1.29 (0.86-1.93) | 0.21 | 1.25 (0.85-1.83) | 0.26 |
|  | **18.5-<25 kg/m^2** | 369 (7.8%) (7.1% - 8.6%) | 252 (10.3%) (9.1% - 11.6%) | 1.00 |  | 1.00 |  |
|  | **25-<30 kg/m^2** | 116 (7.9%) (6.6% - 9.4%) | 89 (10.5%) (8.6% - 12.9%) | 0.89 (0.70-1.14) | 0.36 | 0.94 (0.73-1.21) | 0.63 |
|  | **30-<35 kg/m^2** | 43 (12.7%) (9.3% - 16.7%) | 33 (16.3%) (11.8% - 22.5%) | 1.39 (0.93-2.06) | 0.11 | 1.39 (0.93-2.08) | 0.11 |
|  | **>=35 kg/m^2** | 24 (24.0%) (16.0% - 33.6%) | 17 (25.4%) (16.5% - 38.6%) | 2.78 (1.62-4.76) | 0.0002 | 2.72 (1.55-4.78) | 0.0005 |
| **LDL** | **<2.0 mmol/L** | 79 (6.4%) (5.1% - 7.9%) | 63 (8.4%) (6.7% - 10.8%) | 0.93 (0.66-1.30) | 0.67 | 0.92 (0.66-1.28) | 0.62 |
|  | **2.0-<2.5 mmol/L** | 145 (7.6%) (6.5% - 8.9%) | 110 (9.5%) (8.0% - 11.5%) | 1.00 |  | 1.00 |  |
|  | **2.5-<3.0 mmol/L** | 151 (8.6%) (7.3% - 10.0%) | 113 (10.2%) (8.5% - 12.2%) | 1.17 (0.88-1.54) | 0.28 | 1.10 (0.83-1.45) | 0.51 |
|  | **3.0-<3.5 mmol/L** | 99 (9.1%) (7.5% - 11.0%) | 80 (11.6%) (9.4% - 14.4%) | 1.22 (0.90-1.66) | 0.20 | 1.12 (0.82-1.52) | 0.49 |
|  | **3.5-<4.0 mmol/L** | 61 (11.3%) (8.7% - 14.2%) | 50 (14.0%) (10.8% - 18.3%) | 1.60 (1.13-2.27) | 0.0079 | 1.37 (0.97-1.95) | 0.076 |
|  | **>=4.0 mmol/L** | 43 (15.6%) (11.5% - 20.4%) | 31 (17.6%) (12.7% - 24.6%) | 2.06 (1.29-3.28) | 0.0024 | 1.65 (1.03-2.63) | 0.037 |
| **HDL** | **<1.0 mmol/L** | 54 (12.5%) (9.6% - 16.0%) | 42 (16.2%) (12.2% - 21.6%) | 1.63 (1.09-2.45) | 0.018 | 1.59 (1.07-2.37) | 0.022 |
|  | **1.0-<1.5 mmol/L** | 288 (8.4%) (7.5% - 9.4%) | 206 (10.9%) (9.6% - 12.4%) | 1.00 |  | 1.00 |  |
|  | **1.5-<2.0 mmol/L** | 201 (8.5%) (7.4% - 9.7%) | 146 (10.6%) (9.1% - 12.4%) | 1.02 (0.82-1.27) | 0.85 | 1.07 (0.85-1.33) | 0.57 |
|  | **>=2.0 mmol/L** | 43 (7.7%) (5.6% - 10.2%) | 33 (9.8%) (7.1% - 13.8%) | 0.97 (0.67-1.41) | 0.88 | 1.04 (0.72-1.50) | 0.84 |
| **Triglycerides** | **<0.5 mmol/L** | 18 (11.8%) (7.1% - 18.0%) | 13 (11.9%) (7.1% - 20.4%) | 1.83 (1.02-3.27) | 0.042 | 1.83 (1.00-3.35) | 0.049 |
|  | **0.5-<1.0 mmol/L** | 180 (6.4%) (5.5% - 7.4%) | 131 (8.1%) (6.9% - 9.6%) | 1.00 |  | 1.00 |  |
|  | **1.0-<1.5 mmol/L** | 154 (7.6%) (6.5% - 8.9%) | 119 (9.8%) (8.3% - 11.7%) | 1.14 (0.89-1.46) | 0.30 | 1.04 (0.81-1.33) | 0.76 |
|  | **1.5-2.0 mmol/L** | 84 (10.3%) (8.3% - 12.6%) | 65 (12.3%) (9.7% - 15.5%) | 1.62 (1.19-2.21) | 0.0023 | 1.32 (0.96-1.82) | 0.089 |
|  | **>=2.0 mmol/L** | 129 (16.9%) (14.3% - 19.8%) | 93 (20.5%) (17.1% - 24.7%) | 2.75 (2.01-3.76) | <.0001 | 2.07 (1.50-2.85) | <.0001 |
| **Cholesterol** | **<4.0 mmol/L** | 84 (5.5%) (4.4% - 6.7%) | 69 (7.9%) (6.3% - 10.0%) | 0.77 (0.58-1.02) | 0.068 | 0.79 (0.59-1.04) | 0.095 |
|  | **4.0-<5.0 mmol/L** | 257 (7.7%) (6.8% - 8.7%) | 183 (9.7%) (8.5% - 11.2%) | 1.00 |  | 1.00 |  |
|  | **5.0-<6.0 mmol/L** | 186 (11.7%) (10.1% - 13.3%) | 141 (14.7%) (12.6% - 17.2%) | 1.67 (1.33-2.10) | <.0001 | 1.50 (1.20-1.89) | 0.0005 |
|  | **>=6.0 mmol/L** | 65 (15.5%) (12.2% - 19.3%) | 45 (17.6%) (13.5% - 23.2%) | 2.37 (1.66-3.37) | <.0001 | 1.95 (1.37-2.79) | 0.0002 |
| **Smoking** | **No** | 391 (8.1%) (7.3% - 8.9%) | 274 (10.8%) (9.6% - 12.1%) | 1.00 |  | 1.00 |  |
|  | **Yes** | 208 (10.1%) (8.8% - 11.4%) | 148 (12.9%) (11.1% - 15.1%) | 1.26 (1.03-1.56) | 0.027 | 1.07 (0.87-1.33) | 0.51 |

## **Supplemental Table 4.** Patient characteristics (all patients 13 years of age or later at diabetes diagnosis)

| **Variable** | **All patients (n=4606)** | **All patients with no albuminuria (n=4260)** | **All patients with micro- or macroalbuminuria in any time period (n=346)** | **All patients with no macroalbuminuria (n=4536)** | **All patients with macroalbuminuria in any time period (n=70)** |
| --- | --- | --- | --- | --- | --- |
| **Sex** |  |  |  |  |  |
| **Male** | 2823 (61.3%) | 2634 (61.8%) | 189 (54.6%) | 2787 (61.4%) | 36 (51.4%) |
| **Female** | 1783 (38.7%) | 1626 (38.2%) | 157 (45.4%) | 1749 (38.6%) | 34 (48.6%) |
| **Age at diagnosis** | 20.2 (4.8) 19.7 (13.0; 29.4) n=4606 | 20.2 (4.8) 19.7 (13.0; 29.4) n=4260 | 20.8 (5.0) 20.7 (13.0; 29.4) n=346 | 20.2 (4.8) 19.7 (13.0; 29.4) n=4536 | 21.5 (5.1) 21.6 (13.1; 29.2) n=70 |
| **Age at first visit** | 21.9 (5.3) 22.0 (13.0; 34.0) n=4606 | 21.9 (5.3) 21.0 (13.0; 34.0) n=4260 | 22.9 (5.6) 23.0 (13.1; 34.0) n=346 | 21.9 (5.3) 22.0 (13.0; 34.0) n=4536 | 23.8 (5.7) 23.5 (13.2; 34.0) n=70 |
| **Diabetes onset year** | 2003 (4) 2003 (1993; 2009) n=4606 | 2003 (4) 2004 (1993; 2009) n=4260 | 2001 (4) 2001 (1993; 2009) n=346 | 2003 (4) 2003 (1993; 2009) n=4536 | 2002 (4) 2002 (1993; 2009) n=70 |
| **HbA1c mean (%) for longest follow-up** | 7.76 (1.13) 7.66 (4.82; 13.59) n=4606 | 7.72 (1.10) 7.62 (4.82; 13.26) n=4260 | 8.26 (1.32) 8.15 (5.39; 13.59) n=346 | 7.76 (1.13) 7.65 (4.82; 13.59) n=4536 | 8.24 (1.20) 8.22 (5.73; 11.50) n=70 |
| **HbA1c mean (mmol/mol) for longest follow-up** | 61.3 (12.3) 60.2 (29.1; 125.0) n=4606 | 60.9 (12.1) 59.8 (29.1; 121.3) n=4260 | 66.7 (14.4) 65.5 (35.4; 125.0) n=346 | 61.2 (12.3) 60.1 (29.1; 125.0) n=4536 | 66.5 (13.2) 66.4 (39.1; 102.2) n=70 |
| **HbA1c mean category for longest follow-up** |  |  |  |  |  |
| **<48 mmol/mol** | 571 (12.4%) | 550 (12.9%) | 21 (6.1%) | 567 (12.5%) | 4 (5.7%) |
| **48-52 mmol/mol** | 614 (13.3%) | 581 (13.6%) | 33 (9.5%) | 607 (13.4%) | 7 (10.0%) |
| **53-57 mmol/mol** | 783 (17.0%) | 730 (17.1%) | 53 (15.3%) | 774 (17.1%) | 9 (12.9%) |
| **58-70 mmol/mol** | 1628 (35.3%) | 1515 (35.6%) | 113 (32.7%) | 1605 (35.4%) | 23 (32.9%) |
| **>70 mmol/mol** | 1010 (21.9%) | 884 (20.8%) | 126 (36.4%) | 983 (21.7%) | 27 (38.6%) |
| **HbA1c SD for longest follow-up** | 9.58 (5.39) 8.36 (0.00; 47.71) n=4605 | 9.47 (5.33) 8.27 (0.00; 47.71) n=4260 | 11.0 (5.9) 9.6 (1.5; 37.3) n=345 | 9.56 (5.37) 8.34 (0.00; 47.71) n=4536 | 11.3 (6.5) 9.6 (1.5; 35.2) n=69 |
| **SBP mean (mmHg) for longest follow-up** | 119.3 (8.7) 118.8 (81.3; 175.0) n=4605 | 119.2 (8.5) 118.8 (81.3; 166.7) n=4259 | 121.4 (10.8) 120.1 (94.0; 175.0) n=346 | 119.3 (8.6) 118.8 (81.3; 166.7) n=4535 | 122.7 (13.5) 120.0 (101.1; 175.0) n=70 |
| **SBP mean category for longest follow-up** |  |  |  |  |  |
| **<110 mmHg** | 582 (12.6%) | 543 (12.7%) | 39 (11.3%) | 573 (12.6%) | 9 (12.9%) |
| **110-<120 mmHg** | 1933 (42.0%) | 1802 (42.3%) | 131 (37.9%) | 1908 (42.1%) | 25 (35.7%) |
| **120-<130 mmHg** | 1577 (34.2%) | 1463 (34.4%) | 114 (32.9%) | 1559 (34.4%) | 18 (25.7%) |
| **130-<140 mmHg** | 428 (9.3%) | 388 (9.1%) | 40 (11.6%) | 419 (9.2%) | 9 (12.9%) |
| **>=140 mmHg** | 85 (1.8%) | 63 (1.5%) | 22 (6.4%) | 76 (1.7%) | 9 (12.9%) |
| **DBP mean (mmHg) for longest follow-up** | 72.2 (5.9) 72.0 (53.1; 105.0) n=4605 | 72.0 (5.7) 71.8 (53.1; 105.0) n=4259 | 74.3 (7.3) 73.3 (54.0; 100.4) n=346 | 72.1 (5.8) 71.9 (53.1; 105.0) n=4535 | 75.4 (9.2) 73.3 (54.0; 95.0) n=70 |
| **DBP mean category for longest follow-up** |  |  |  |  |  |
| **<60 mmHg** | 44 (1.0%) | 41 (1.0%) | 3 (0.9%) | 43 (0.9%) | 1 (1.4%) |
| **60-<70 mmHg** | 1597 (34.7%) | 1505 (35.3%) | 92 (26.6%) | 1576 (34.8%) | 21 (30.0%) |
| **70-<80 mmHg** | 2527 (54.9%) | 2343 (55.0%) | 184 (53.2%) | 2500 (55.1%) | 27 (38.6%) |
| **80-<85 mmHg** | 340 (7.4%) | 301 (7.1%) | 39 (11.3%) | 331 (7.3%) | 9 (12.9%) |
| **>=85 mmHg** | 97 (2.1%) | 69 (1.6%) | 28 (8.1%) | 85 (1.9%) | 12 (17.1%) |
| **BMI mean (kg/m2) for longest follow-up** | 24.7 (4.0) 24.1 (15.8; 48.4) n=4577 | 24.6 (3.9) 24.0 (15.8; 48.4) n=4235 | 25.7 (5.1) 24.6 (17.0; 44.0) n=342 | 24.6 (4.0) 24.1 (15.8; 48.4) n=4508 | 26.0 (5.2) 24.9 (17.6; 42.0) n=69 |
| **BMI mean category for longest follow-up** |  |  |  |  |  |
| **<18.5 kg/m^2** | 86 (1.9%) | 77 (1.8%) | 9 (2.6%) | 84 (1.9%) | 2 (2.9%) |
| **18.5-<25 kg/m^2** | 2682 (58.6%) | 2505 (59.1%) | 177 (51.8%) | 2649 (58.8%) | 33 (47.8%) |
| **25-<30 kg/m^2** | 1408 (30.8%) | 1309 (30.9%) | 99 (28.9%) | 1387 (30.8%) | 21 (30.4%) |
| **30-<35 kg/m^2** | 315 (6.9%) | 279 (6.6%) | 36 (10.5%) | 305 (6.8%) | 10 (14.5%) |
| **>=35 kg/m^2** | 86 (1.9%) | 65 (1.5%) | 21 (6.1%) | 83 (1.8%) | 3 (4.3%) |
| **HDL mean (mmol/L) for longest follow-up** | 1.51 (0.37) 1.47 (0.53; 4.80) n=4447 | 1.51 (0.37) 1.47 (0.53; 4.80) n=4113 | 1.44 (0.39) 1.40 (0.58; 3.70) n=334 | 1.51 (0.37) 1.47 (0.53; 4.80) n=4381 | 1.45 (0.48) 1.40 (0.60; 3.70) n=66 |
| **HDL mean category for longest follow-up** |  |  |  |  |  |
| **<1.0 mmol/L** | 216 (4.9%) | 184 (4.5%) | 32 (9.6%) | 207 (4.7%) | 9 (13.6%) |
| **1.0-<1.5 mmol/L** | 2128 (47.9%) | 1961 (47.7%) | 167 (50.0%) | 2098 (47.9%) | 30 (45.5%) |
| **1.5-<2.0 mmol/L** | 1662 (37.4%) | 1555 (37.8%) | 107 (32.0%) | 1643 (37.5%) | 19 (28.8%) |
| **>=2.0 mmol/L** | 441 (9.9%) | 413 (10.0%) | 28 (8.4%) | 433 (9.9%) | 8 (12.1%) |
| **LDL mean (mmol/L) for longest follow-up** | 2.58 (0.68) 2.52 (0.73; 8.94) n=4453 | 2.57 (0.68) 2.52 (0.73; 8.94) n=4119 | 2.67 (0.70) 2.64 (1.00; 6.11) n=334 | 2.58 (0.68) 2.52 (0.73; 8.94) n=4390 | 2.63 (0.82) 2.57 (1.00; 6.11) n=63 |
| **LDL mean category for longest follow-up** |  |  |  |  |  |
| **<2.0 mmol/L** | 828 (18.6%) | 773 (18.8%) | 55 (16.5%) | 817 (18.6%) | 11 (17.5%) |
| **2.0-<2.5 mmol/L** | 1341 (30.1%) | 1248 (30.3%) | 93 (27.8%) | 1322 (30.1%) | 19 (30.2%) |
| **2.5-<3.0 mmol/L** | 1202 (27.0%) | 1122 (27.2%) | 80 (24.0%) | 1186 (27.0%) | 16 (25.4%) |
| **3.0-<3.5 mmol/L** | 687 (15.4%) | 622 (15.1%) | 65 (19.5%) | 677 (15.4%) | 10 (15.9%) |
| **3.5-<4.0 mmol/L** | 274 (6.2%) | 244 (5.9%) | 30 (9.0%) | 270 (6.2%) | 4 (6.3%) |
| **>=4.0 mmol/L** | 121 (2.7%) | 110 (2.7%) | 11 (3.3%) | 118 (2.7%) | 3 (4.8%) |
| **Cholesterol mean (mmol/L) for longest follow-up** | 4.55 (0.78) 4.50 (2.27; 11.00) n=4473 | 4.53 (0.78) 4.48 (2.27; 11.00) n=4138 | 4.70 (0.84) 4.63 (2.80; 8.73) n=335 | 4.54 (0.78) 4.50 (2.27; 11.00) n=4407 | 4.82 (1.04) 4.65 (3.27; 8.73) n=66 |
| **Cholesterol mean category for longest follow-up** |  |  |  |  |  |
| **<4.0 mmol/L** | 1047 (23.4%) | 977 (23.6%) | 70 (20.9%) | 1034 (23.5%) | 13 (19.7%) |
| **4.0-<5.0 mmol/L** | 2256 (50.4%) | 2111 (51.0%) | 145 (43.3%) | 2227 (50.5%) | 29 (43.9%) |
| **5.0-<6.0 mmol/L** | 985 (22.0%) | 886 (21.4%) | 99 (29.6%) | 967 (21.9%) | 18 (27.3%) |
| **>=6.0 mmol/L** | 185 (4.1%) | 164 (4.0%) | 21 (6.3%) | 179 (4.1%) | 6 (9.1%) |
| **Triglycerides mean (mmol/L) for longest follow-up** | 1.08 (0.74) 0.90 (0.20; 18.06) n=4405 | 1.05 (0.63) 0.89 (0.20; 9.62) n=4076 | 1.44 (1.51) 1.03 (0.37; 18.06) n=329 | 1.06 (0.68) 0.90 (0.20; 11.50) n=4343 | 1.93 (2.44) 1.22 (0.40; 18.06) n=62 |
| **Triglycerides mean category for longest follow-up** |  |  |  |  |  |
| **<0.5 mmol/L** | 180 (4.1%) | 174 (4.3%) | 6 (1.8%) | 179 (4.1%) | 1 (1.6%) |
| **0.5-<1.0 mmol/L** | 2425 (55.1%) | 2280 (55.9%) | 145 (44.1%) | 2401 (55.3%) | 24 (38.7%) |
| **1.0-<1.5 mmol/L** | 1134 (25.7%) | 1042 (25.6%) | 92 (28.0%) | 1124 (25.9%) | 10 (16.1%) |
| **1.5-2.0 mmol/L** | 369 (8.4%) | 335 (8.2%) | 34 (10.3%) | 362 (8.3%) | 7 (11.3%) |
| **>=2.0 mmol/L** | 297 (6.7%) | 245 (6.0%) | 52 (15.8%) | 277 (6.4%) | 20 (32.3%) |
| **Smoking at any time before for longest follow-up** |  |  |  |  |  |
| **No** | 3552 (77.2%) | 3308 (77.7%) | 244 (70.5%) | 3505 (77.4%) | 47 (67.1%) |
| **Yes** | 1049 (22.8%) | 947 (22.3%) | 102 (29.5%) | 1026 (22.6%) | 23 (32.9%) |
| For categorical variables n (%) is presented. For continuous variables Mean (SD) / Median (Min; Max) / n= is presented. | | | | | |

## **Supplemental Table 5.** Generalized Estimating Equation (GEE) models for the impact of various variables at any time before on albuminuria endpoints (all patients 13 years of age or later at diabetes diagnosis)

|  | | | | **Adjusted for age and sex** | | **Adjusted for age, sex and HbA1c mean** | |
| --- | --- | --- | --- | --- | --- | --- | --- |
| **Variable** | **Category** | **n (%) (95% CI) events** | **n (%) (95% CI) persons with events** | **OR (95% CI)** | **p-value** | **OR (95% CI)** | **p-value** |
| **Microalbuminuria/Macroalbuminuria vs None** | | | | | | | |
| **SBP** | **<110 mmHg** | 49 (5.5%) (4.1% - 7.3%) | 41 (6.7%) (5.0% - 9.1%) | 0.88 (0.62-1.26) | 0.50 | 0.87 (0.61-1.26) | 0.46 |
|  | **110-<120 mmHg** | 174 (5.9%) (5.1% - 6.8%) | 139 (6.9%) (5.9% - 8.1%) | 1.00 |  | 1.00 |  |
|  | **120-<130 mmHg** | 138 (6.0%) (5.1% - 7.0%) | 112 (6.8%) (5.7% - 8.2%) | 0.97 (0.72-1.32) | 0.86 | 0.98 (0.73-1.32) | 0.89 |
|  | **130-<140 mmHg** | 54 (8.8%) (6.6% - 11.3%) | 42 (9.4%) (7.0% - 12.7%) | 1.43 (0.96-2.12) | 0.079 | 1.41 (0.95-2.09) | 0.084 |
|  | **>=140 mmHg** | 25 (21.6%) (14.5% - 30.1%) | 22 (25.0%) (17.1% - 36.2%) | 3.92 (2.34-6.57) | <.0001 | 3.82 (2.27-6.43) | <.0001 |
| **DBP** | **<60 mmHg** | 3 (4.5%) (0.9% - 12.5%) | 3 (6.0%) (2.2% - 18.9%) | 0.79 (0.13-4.72) | 0.79 | 0.90 (0.15-5.53) | 0.91 |
|  | **60-<70 mmHg** | 117 (4.7%) (3.9% - 5.6%) | 93 (5.5%) (4.6% - 6.8%) | 1.00 |  | 1.00 |  |
|  | **70-<80 mmHg** | 238 (6.4%) (5.7% - 7.3%) | 187 (7.3%) (6.3% - 8.4%) | 1.32 (1.02-1.71) | 0.036 | 1.23 (0.95-1.60) | 0.12 |
|  | **80-<85 mmHg** | 48 (9.9%) (7.4% - 12.9%) | 39 (10.7%) (7.9% - 14.5%) | 1.77 (1.15-2.74) | 0.0098 | 1.55 (1.01-2.36) | 0.044 |
|  | **>=85 mmHg** | 34 (26.8%) (19.3% - 35.4%) | 29 (28.4%) (20.6% - 38.9%) | 5.96 (3.69-9.64) | <.0001 | 5.25 (3.21-8.57) | <.0001 |
| **BMI** | **<18.5 kg/m^2** | 13 (8.4%) (4.6% - 14.0%) | 9 (9.8%) (5.3% - 18.9%) | 1.00 (0.18-5.52) | 1.00 | 0.97 (0.23-4.03) | 0.97 |
|  | **18.5-<25 kg/m^2** | 224 (5.5%) (4.8% - 6.3%) | 179 (6.6%) (5.7% - 7.6%) | 1.00 |  | 1.00 |  |
|  | **25-<30 kg/m^2** | 126 (6.1%) (5.1% - 7.3%) | 99 (7.0%) (5.7% - 8.5%) | 1.01 (0.79-1.30) | 0.92 | 0.98 (0.76-1.26) | 0.85 |
|  | **30-<35 kg/m^2** | 44 (10.0%) (7.4% - 13.2%) | 36 (11.3%) (8.3% - 15.6%) | 1.75 (1.21-2.54) | 0.0032 | 1.48 (1.01-2.17) | 0.042 |
|  | **>=35 kg/m^2** | 28 (24.3%) (16.8% - 33.2%) | 21 (24.1%) (16.4% - 35.4%) | 4.11 (2.49-6.78) | <.0001 | 3.13 (1.86-5.26) | <.0001 |
| **LDL** | **<2.0 mmol/L** | 70 (5.5%) (4.3% - 6.9%) | 54 (6.2%) (4.8% - 8.1%) | 0.94 (0.68-1.29) | 0.69 | 0.96 (0.70-1.33) | 0.82 |
|  | **2.0-<2.5 mmol/L** | 122 (6.1%) (5.1% - 7.3%) | 99 (7.0%) (5.8% - 8.5%) | 1.00 |  | 1.00 |  |
|  | **2.5-<3.0 mmol/L** | 102 (5.7%) (4.7% - 6.9%) | 80 (6.3%) (5.1% - 7.9%) | 0.93 (0.69-1.26) | 0.66 | 0.87 (0.64-1.16) | 0.34 |
|  | **3.0-<3.5 mmol/L** | 81 (8.0%) (6.4% - 9.9%) | 68 (9.2%) (7.3% - 11.7%) | 1.27 (0.90-1.80) | 0.17 | 1.12 (0.79-1.57) | 0.52 |
|  | **3.5-<4.0 mmol/L** | 34 (8.5%) (6.0% - 11.7%) | 31 (10.3%) (7.3% - 14.6%) | 1.45 (0.95-2.22) | 0.084 | 1.20 (0.78-1.83) | 0.40 |
|  | **>=4.0 mmol/L** | 16 (8.6%) (5.0% - 13.6%) | 13 (9.8%) (5.8% - 16.9%) | 1.11 (0.46-2.71) | 0.82 | 0.90 (0.41-1.98) | 0.79 |
| **HDL** | **<1.0 mmol/L** | 41 (12.9%) (9.4% - 17.1%) | 32 (14.3%) (10.3% - 20.0%) | 1.93 (1.25-2.99) | 0.0030 | 1.68 (1.09-2.59) | 0.019 |
|  | **1.0-<1.5 mmol/L** | 209 (6.6%) (5.8% - 7.5%) | 167 (7.6%) (6.6% - 8.9%) | 1.00 |  | 1.00 |  |
|  | **1.5-<2.0 mmol/L** | 134 (5.4%) (4.5% - 6.3%) | 106 (6.2%) (5.1% - 7.5%) | 0.68 (0.53-0.87) | 0.0022 | 0.73 (0.57-0.93) | 0.013 |
|  | **>=2.0 mmol/L** | 40 (6.1%) (4.4% - 8.2%) | 31 (6.8%) (4.8% - 9.7%) | 0.79 (0.53-1.18) | 0.25 | 0.90 (0.60-1.35) | 0.60 |
| **Triglycerides** | **<0.5 mmol/L** | 11 (3.9%) (1.9% - 6.8%) | 10 (5.0%) (2.7% - 9.5%) | 0.76 (0.34-1.67) | 0.49 | 0.79 (0.36-1.72) | 0.55 |
|  | **0.5-<1.0 mmol/L** | 182 (5.0%) (4.3% - 5.8%) | 142 (5.7%) (4.9% - 6.8%) | 1.00 |  | 1.00 |  |
|  | **1.0-<1.5 mmol/L** | 113 (6.8%) (5.6% - 8.1%) | 92 (7.7%) (6.3% - 9.5%) | 1.45 (1.10-1.90) | 0.0085 | 1.27 (0.96-1.68) | 0.094 |
|  | **1.5-2.0 mmol/L** | 45 (8.5%) (6.2% - 11.2%) | 38 (9.5%) (7.0% - 13.0%) | 2.08 (1.43-3.03) | 0.0001 | 1.63 (1.11-2.41) | 0.013 |
|  | **>=2.0 mmol/L** | 67 (14.9%) (11.7% - 18.5%) | 55 (17.5%) (13.7% - 22.5%) | 3.43 (2.39-4.92) | <.0001 | 2.49 (1.71-3.62) | <.0001 |
| **Cholesterol** | **<4.0 mmol/L** | 88 (5.5%) (4.5% - 6.8%) | 69 (6.3%) (5.0% - 8.1%) | 1.09 (0.81-1.47) | 0.55 | 1.14 (0.85-1.54) | 0.38 |
|  | **4.0-<5.0 mmol/L** | 184 (5.5%) (4.7% - 6.3%) | 147 (6.3%) (5.4% - 7.5%) | 1.00 |  | 1.00 |  |
|  | **5.0-<6.0 mmol/L** | 124 (8.6%) (7.2% - 10.2%) | 100 (9.7%) (8.1% - 11.8%) | 1.65 (1.27-2.14) | 0.0002 | 1.42 (1.09-1.85) | 0.010 |
|  | **>=6.0 mmol/L** | 29 (10.5%) (7.1% - 14.7%) | 23 (11.9%) (8.1% - 17.8%) | 2.13 (1.30-3.47) | 0.0025 | 1.69 (1.04-2.74) | 0.034 |
| **Smoking** | **No** | 323 (6.0%) (5.4% - 6.7%) | 245 (6.8%) (6.0% - 7.7%) | 1.00 |  | 1.00 |  |
|  | **Yes** | 117 (7.8%) (6.5% - 9.2%) | 101 (9.6%) (8.0% - 11.7%) | 1.43 (1.12-1.83) | 0.0046 | 1.11 (0.86-1.42) | 0.44 |
| **Macroalbuminuria vs None/Microalbuminuria** | | | | | | | |
| **SBP** | **<110 mmHg** | 10 (1.1%) (0.5% - 2.1%) | 9 (1.5%) (0.8% - 3.0%) | 0.94 (0.41-2.15) | 0.89 | 0.95 (0.41-2.17) | 0.90 |
|  | **110-<120 mmHg** | 30 (1.0%) (0.7% - 1.5%) | 27 (1.3%) (0.9% - 2.0%) | 1.00 |  | 1.00 |  |
|  | **120-<130 mmHg** | 21 (0.9%) (0.6% - 1.4%) | 19 (1.2%) (0.7% - 1.9%) | 0.95 (0.50-1.79) | 0.87 | 0.93 (0.50-1.75) | 0.83 |
|  | **130-<140 mmHg** | 10 (1.6%) (0.8% - 3.0%) | 9 (2.0%) (1.1% - 4.1%) | 1.82 (0.82-4.01) | 0.14 | 1.73 (0.79-3.80) | 0.17 |
|  | **>=140 mmHg** | 10 (8.6%) (4.2% - 15.3%) | 9 (10.2%) (5.5% - 19.7%) | 9.58 (4.33-21.19) | <.0001 | 8.66 (3.88-19.33) | <.0001 |
| **DBP** | **<60 mmHg** | 1 (1.5%) (0.0% - 8.0%) | 1 (2.0%) (0.5% - 13.5%) | 1.52 (0.20-11.53) | 0.69 | 1.67 (0.22-12.67) | 0.62 |
|  | **60-<70 mmHg** | 25 (1.0%) (0.7% - 1.5%) | 22 (1.3%) (0.9% - 2.1%) | 1.00 |  | 1.00 |  |
|  | **70-<80 mmHg** | 32 (0.9%) (0.6% - 1.2%) | 27 (1.1%) (0.7% - 1.6%) | 0.86 (0.45-1.62) | 0.64 | 0.80 (0.43-1.52) | 0.50 |
|  | **80-<85 mmHg** | 9 (1.9%) (0.9% - 3.5%) | 8 (2.2%) (1.1% - 4.6%) | 1.73 (0.68-4.40) | 0.25 | 1.48 (0.60-3.65) | 0.40 |
|  | **>=85 mmHg** | 14 (11.0%) (6.2% - 17.8%) | 13 (12.7%) (7.6% - 21.8%) | 10.53 (4.86-22.78) | <.0001 | 9.04 (4.17-19.60) | <.0001 |
| **BMI** | **<18.5 kg/m^2** | 2 (1.3%) (0.2% - 4.6%) | 2 (2.2%) (0.7% - 9.1%) | 1.50 (0.35-6.52) | 0.59 | 1.33 (0.30-5.81) | 0.71 |
|  | **18.5-<25 kg/m^2** | 42 (1.0%) (0.7% - 1.4%) | 34 (1.3%) (0.9% - 1.8%) | 1.00 |  | 1.00 |  |
|  | **25-<30 kg/m^2** | 23 (1.1%) (0.7% - 1.7%) | 21 (1.5%) (1.0% - 2.3%) | 1.00 (0.57-1.75) | 1.00 | 0.96 (0.55-1.68) | 0.89 |
|  | **30-<35 kg/m^2** | 10 (2.3%) (1.1% - 4.1%) | 10 (3.1%) (1.7% - 6.1%) | 1.93 (0.95-3.94) | 0.070 | 1.66 (0.81-3.41) | 0.17 |
|  | **>=35 kg/m^2** | 3 (2.6%) (0.5% - 7.4%) | 3 (3.4%) (1.3% - 11.2%) | 1.93 (0.57-6.50) | 0.29 | 1.50 (0.44-5.09) | 0.51 |
| **LDL** | **<2.0 mmol/L** | 15 (1.2%) (0.7% - 1.9%) | 11 (1.3%) (0.7% - 2.4%) | 1.14 (0.52-2.48) | 0.75 | 1.15 (0.53-2.52) | 0.72 |
|  | **2.0-<2.5 mmol/L** | 22 (1.1%) (0.7% - 1.7%) | 20 (1.4%) (0.9% - 2.2%) | 1.00 |  | 1.00 |  |
|  | **2.5-<3.0 mmol/L** | 17 (1.0%) (0.6% - 1.5%) | 16 (1.3%) (0.8% - 2.1%) | 0.84 (0.43-1.62) | 0.60 | 0.80 (0.41-1.55) | 0.51 |
|  | **3.0-<3.5 mmol/L** | 10 (1.0%) (0.5% - 1.8%) | 10 (1.4%) (0.7% - 2.7%) | 0.84 (0.40-1.77) | 0.65 | 0.76 (0.36-1.59) | 0.47 |
|  | **3.5-<4.0 mmol/L** | 5 (1.3%) (0.4% - 2.9%) | 5 (1.7%) (0.7% - 4.3%) | 1.07 (0.39-2.90) | 0.89 | 0.94 (0.35-2.52) | 0.90 |
|  | **>=4.0 mmol/L** | 4 (2.2%) (0.6% - 5.4%) | 3 (2.3%) (0.8% - 7.5%) | 1.91 (0.50-7.23) | 0.34 | 1.56 (0.42-5.76) | 0.50 |
| **HDL** | **<1.0 mmol/L** | 10 (3.2%) (1.5% - 5.7%) | 9 (4.0%) (2.2% - 8.0%) | 3.03 (1.37-6.70) | 0.0061 | 2.67 (1.17-6.10) | 0.020 |
|  | **1.0-<1.5 mmol/L** | 34 (1.1%) (0.7% - 1.5%) | 30 (1.4%) (1.0% - 2.0%) | 1.00 |  | 1.00 |  |
|  | **1.5-<2.0 mmol/L** | 23 (0.9%) (0.6% - 1.4%) | 19 (1.1%) (0.7% - 1.8%) | 0.75 (0.41-1.38) | 0.35 | 0.79 (0.43-1.45) | 0.44 |
|  | **>=2.0 mmol/L** | 9 (1.4%) (0.6% - 2.6%) | 8 (1.7%) (0.9% - 3.7%) | 0.99 (0.40-2.45) | 0.99 | 1.10 (0.45-2.68) | 0.84 |
| **Triglycerides** | **<0.5 mmol/L** | 2 (0.7%) (0.1% - 2.5%) | 1 (0.5%) (0.1% - 3.5%) | 0.88 (0.12-6.54) | 0.90 | 0.90 (0.12-6.73) | 0.92 |
|  | **0.5-<1.0 mmol/L** | 27 (0.7%) (0.5% - 1.1%) | 24 (1.0%) (0.7% - 1.5%) | 1.00 |  | 1.00 |  |
|  | **1.0-<1.5 mmol/L** | 13 (0.8%) (0.4% - 1.3%) | 10 (0.8%) (0.5% - 1.6%) | 1.08 (0.50-2.36) | 0.84 | 1.04 (0.46-2.34) | 0.93 |
|  | **1.5-2.0 mmol/L** | 7 (1.3%) (0.5% - 2.7%) | 7 (1.7%) (0.9% - 3.9%) | 1.93 (0.81-4.61) | 0.14 | 1.79 (0.73-4.34) | 0.20 |
|  | **>=2.0 mmol/L** | 22 (4.9%) (3.1% - 7.3%) | 20 (6.4%) (4.2% - 10.0%) | 7.47 (3.93-14.17) | <.0001 | 6.65 (3.46-12.79) | <.0001 |
| **Cholesterol** | **<4.0 mmol/L** | 17 (1.1%) (0.6% - 1.7%) | 13 (1.2%) (0.7% - 2.1%) | 1.24 (0.64-2.42) | 0.52 | 1.26 (0.65-2.46) | 0.49 |
|  | **4.0-<5.0 mmol/L** | 33 (1.0%) (0.7% - 1.4%) | 30 (1.3%) (0.9% - 1.9%) | 1.00 |  | 1.00 |  |
|  | **5.0-<6.0 mmol/L** | 18 (1.3%) (0.7% - 2.0%) | 18 (1.8%) (1.1% - 2.9%) | 1.18 (0.66-2.12) | 0.58 | 1.06 (0.58-1.91) | 0.86 |
|  | **>=6.0 mmol/L** | 8 (2.9%) (1.3% - 5.6%) | 7 (3.6%) (1.8% - 8.0%) | 2.96 (1.22-7.18) | 0.016 | 2.50 (1.04-5.99) | 0.040 |
| **Smoking** | **No** | 55 (1.0%) (0.8% - 1.3%) | 47 (1.3%) (1.0% - 1.8%) | 1.00 |  | 1.00 |  |
|  | **Yes** | 26 (1.7%) (1.1% - 2.5%) | 23 (2.2%) (1.5% - 3.4%) | 1.78 (1.07-2.95) | 0.027 | 1.48 (0.86-2.55) | 0.15 |

## **Supplemental Table 6.** Generalized Estimating Equation (GEE) models for the impact of risk factors on nephropathy endpoints (all patients 13 years of age or later at diabetes diagnosis).

|  |  |  | **Adjusted for age and sex** | | | **Multivariable adjusted*** | | |
| --- | --- | --- | --- | --- | --- | --- | --- | --- |
| **Variable** | **n (%) (95% CI) events** | **n (%) (95% CI) persons with events** | **OR (95% CI) per specified unit increase** | **OR (95% CI) per 1 SD increase** | **p-value** | **OR (95% CI) per specified unit increase** | **OR (95% CI) per 1 SD increase** | **p-value** |
| **Microalbuminuria/Macroalbuminuria vs None** | | | | | | | | |
| **Mean HbA1c (mmol/mol) (by 10 unit increase)** | 440 (6.4%) (5.8% - 7.0%) | 346 (7.5%) (6.8% - 8.3%) | 1.43 (1.32-1.56) | 1.56 (1.41-1.72) | <.0001 | 1.36 (1.23-1.50) | 1.46 (1.30-1.65) | <.0001 |
| **Mean HbA1c (%) (by 1 unit increase)** | 440 (6.4%) (5.8% - 7.0%) | 346 (7.5%) (6.8% - 8.3%) | 1.48 (1.35-1.62) | 1.56 (1.41-1.73) | <.0001 | 1.40 (1.26-1.56) | 1.46 (1.30-1.65) | <.0001 |
| **HbA1c SD (mmol/mol) (by 1 unit increase)** | 439 (6.4%) (5.8% - 7.0%) | 345 (7.5%) (6.7% - 8.3%) | 1.06 (1.04-1.08) | 1.38 (1.26-1.51) | <.0001 | 1.05 (1.03-1.07) | 1.27 (1.15-1.40) | <.0001 |
| **Mean SBP (mmHg) (by 10 unit increase)** | 440 (6.4%) (5.8% - 7.0%) | 346 (7.5%) (6.8% - 8.3%) | 1.44 (1.25-1.65) | 1.37 (1.21-1.55) | <.0001 | 1.41 (1.23-1.63) | 1.35 (1.20-1.53) | <.0001 |
| **Mean DBP (mmHg) (by 5 unit increase)** | 440 (6.4%) (5.8% - 7.0%) | 346 (7.5%) (6.8% - 8.3%) | 1.38 (1.24-1.53) | 1.45 (1.28-1.64) | <.0001 | 1.32 (1.19-1.47) | 1.38 (1.22-1.56) | <.0001 |
| **Mean BMI (kg/m2) (by 5 unit increase)** | 435 (6.4%) (5.8% - 7.0%) | 342 (7.5%) (6.7% - 8.3%) | 1.32 (1.15-1.51) | 1.24 (1.11-1.38) | <.0001 | 1.24 (1.08-1.42) | 1.18 (1.06-1.31) | 0.0025 |
| **Mean LDL (mmol/L) (by 1 unit increase)** | 425 (6.4%) (5.8% - 7.0%) | 333 (7.5%) (6.7% - 8.3%) | 1.21 (1.02-1.42) | 1.14 (1.02-1.27) | 0.025 | 1.08 (0.92-1.27) | 1.05 (0.94-1.18) | 0.36 |
| **Mean HDL (mmol/L) (by 1 unit increase)** | 424 (6.4%) (5.8% - 7.0%) | 333 (7.5%) (6.7% - 8.3%) | 0.47 (0.31-0.72) | 0.76 (0.65-0.88) | 0.0004 | 0.58 (0.39-0.88) | 0.82 (0.70-0.95) | 0.0097 |
| **Mean triglycerides (mmol/L) (by 1 unit increase)** | 418 (6.4%) (5.8% - 7.0%) | 328 (7.4%) (6.7% - 8.3%) | 1.54 (1.36-1.75) | 1.37 (1.25-1.50) | <.0001 | 1.39 (1.23-1.58) | 1.27 (1.16-1.39) | <.0001 |
| **Mean cholesterol (mmol/L) (by 1 unit increase)** | 425 (6.4%) (5.8% - 7.0%) | 334 (7.5%) (6.7% - 8.3%) | 1.28 (1.11-1.48) | 1.22 (1.09-1.36) | 0.0007 | 1.14 (0.99-1.31) | 1.11 (0.99-1.24) | 0.070 |
| **Smoking at any time before (yes vs no)** | 440 (6.4%) (5.8% - 7.0%) | 346 (7.5%) (6.8% - 8.3%) | 1.43 (1.12-1.83) | 1.16 (1.05-1.28) | 0.0046 | 1.11 (0.86-1.42) | 1.04 (0.94-1.15) | 0.44 |
| **Macroalbuminuria vs None/Microalbuminuria** | | | | | | | | |
| **Mean HbA1c (mmol/mol) (by 10 unit increase)** | 81 (1.2%) (0.9% - 1.5%) | 70 (1.5%) (1.2% - 1.9%) | 1.34 (1.14-1.59) | 1.44 (1.17-1.76) | 0.0004 | 1.11 (0.90-1.36) | 1.13 (0.88-1.47) | 0.34 |
| **Mean HbA1c (%) (by 1 unit increase)** | 81 (1.2%) (0.9% - 1.5%) | 70 (1.5%) (1.2% - 1.9%) | 1.38 (1.15-1.66) | 1.44 (1.18-1.77) | 0.0004 | 1.12 (0.89-1.40) | 1.13 (0.88-1.47) | 0.34 |
| **HbA1c SD (mmol/mol) (by 1 unit increase)** | 80 (1.2%) (0.9% - 1.4%) | 69 (1.5%) (1.2% - 1.9%) | 1.07 (1.03-1.10) | 1.42 (1.19-1.69) | 0.0001 | 1.02 (0.97-1.07) | 1.11 (0.87-1.41) | 0.41 |
| **Mean SBP (mmHg) (by 10 unit increase)** | 81 (1.2%) (0.9% - 1.5%) | 70 (1.5%) (1.2% - 1.9%) | 1.66 (1.25-2.20) | 1.55 (1.21-1.98) | 0.0005 | 1.61 (1.22-2.14) | 1.52 (1.19-1.94) | 0.0009 |
| **Mean DBP (mmHg) (by 5 unit increase)** | 81 (1.2%) (0.9% - 1.5%) | 70 (1.5%) (1.2% - 1.9%) | 1.48 (1.15-1.89) | 1.57 (1.18-2.09) | 0.0019 | 1.42 (1.11-1.82) | 1.50 (1.13-2.00) | 0.0053 |
| **Mean BMI (kg/m2) (by 5 unit increase)** | 80 (1.2%) (0.9% - 1.5%) | 69 (1.5%) (1.2% - 1.9%) | 1.28 (0.98-1.66) | 1.21 (0.99-1.48) | 0.069 | 1.20 (0.93-1.56) | 1.16 (0.94-1.41) | 0.16 |
| **Mean LDL (mmol/L) (by 1 unit increase)** | 73 (1.1%) (0.9% - 1.4%) | 63 (1.4%) (1.1% - 1.8%) | 1.06 (0.69-1.63) | 1.04 (0.77-1.40) | 0.81 | 0.98 (0.66-1.47) | 0.99 (0.75-1.30) | 0.94 |
| **Mean HDL (mmol/L) (by 1 unit increase)** | 76 (1.1%) (0.9% - 1.4%) | 66 (1.5%) (1.1% - 1.9%) | 0.41 (0.15-1.10) | 0.72 (0.50-1.04) | 0.077 | 0.47 (0.18-1.24) | 0.76 (0.53-1.08) | 0.13 |
| **Mean triglycerides (mmol/L) (by 1 unit increase)** | 71 (1.1%) (0.8% - 1.4%) | 62 (1.4%) (1.1% - 1.8%) | 1.61 (1.38-1.87) | 1.41 (1.26-1.57) | <.0001 | 1.55 (1.33-1.81) | 1.37 (1.23-1.54) | <.0001 |
| **Mean cholesterol (mmol/L) (by 1 unit increase)** | 76 (1.1%) (0.9% - 1.4%) | 66 (1.5%) (1.1% - 1.9%) | 1.38 (0.99-1.93) | 1.29 (0.99-1.68) | 0.056 | 1.28 (0.94-1.75) | 1.22 (0.95-1.56) | 0.12 |
| **Smoking at any time before (yes vs no)** | 81 (1.2%) (0.9% - 1.5%) | 70 (1.5%) (1.2% - 1.9%) | 1.78 (1.07-2.95) | 1.27 (1.03-1.56) | 0.027 | 1.48 (0.86-2.55) | 1.18 (0.94-1.47) | 0.15 |
| * HbA1c mean and HbA1c SD adjusted for age, sex, SBP, BMI, triglycerides, cholesterol and smoking. All other variables adjusted for age, sex and HbA1c mean. | | | | | | | | |
